# Genome-Wide Identification and Expression Profiling of *KCS* Gene Family in Passion Fruit (*Passiflora edulis*) Under *Fusarium kyushuense* and Drought Stress Conditions

**DOI:** 10.3389/fpls.2022.872263

**Published:** 2022-04-25

**Authors:** Hafiz Muhammad Rizwan, Fang Shaozhong, Xiaoting Li, Muhammad Bilal Arshad, Ahmed Fathy Yousef, Yang Chenglong, Meng Shi, Mohammed Y. M. Jaber, Muhammad Anwar, Shuai-Ya Hu, Qiang Yang, Kaiwei Sun, Mohamed A. A. Ahmed, Zheng Min, Ralf Oelmüller, Lin Zhimin, Faxing Chen

**Affiliations:** ^1^College of Horticulture, Fujian Agriculture and Forestry University, Fuzhou, China; ^2^Institute of Biotechnology, Fujian Academy of Agricultural Sciences, Fuzhou, China; ^3^Department of Plant Breeding and Genetics, College of Agriculture, University of Sargodha, Sargodha, Pakistan; ^4^Department of Horticulture, College of Agriculture, University of Al-Azhar, Assiut, Egypt; ^5^Department of Plant Production and Protection, Faculty of Agriculture and Veterinary Medicine, An-Najah National University, Nablus, Palestine; ^6^Guangdong Technology Research Center for Marine Algal Bioengineering, Guangdong Key Laboratory of Plant Epigenetics, College of Life Sciences and Oceanography, Shenzhen University, Shenzhen, China; ^7^College of Horticulture, Academy for Advanced Interdisciplinary Studies, Nanjing Agriculture University, Nanjing, China; ^8^Plant Production Department (Horticulture-Medicinal and Aromatic Plants), Faculty of Agriculture (Saba Basha), Alexandria University, Alexandria, Egypt; ^9^Department of Horticulture, Fujian Agricultural Vocational College, Fuzhou, China; ^10^Matthias Schleiden Institute, Plant Physiology, Friedrich-Schiller-University Jena, Jena, Germany

**Keywords:** very long chain fatty acids, wax biosynthesis, synteny, transcription factor, micro-RNA, gene anthology, stress conditions

## Abstract

Plant and fruit surfaces are covered with cuticle wax and provide a protective barrier against biotic and abiotic stresses. Cuticle wax consists of very-long-chain fatty acids (VLCFAs) and their derivatives. β-Ketoacyl-CoA synthase (*KCS*) is a key enzyme in the synthesis of VLCFAs and provides a precursor for the synthesis of cuticle wax, but the *KCS* gene family was yet to be reported in the passion fruit (*Passiflora edulis*). In this study, thirty-two *KCS* genes were identified in the passion fruit genome and phylogenetically grouped as *KCS1*-like, *FAE1*-like, *FDH*-like, and *CER6*-like. Furthermore, thirty-one *PeKCS* genes were positioned on seven chromosomes, while one *PeKCS* was localized to the unassembled genomic scaffold. The *cis*-element analysis provides insight into the possible role of *PeKCS* genes in phytohormones and stress responses. Syntenic analysis revealed that gene duplication played a crucial role in the expansion of the *PeKCS* gene family and underwent a strong purifying selection. All PeKCS proteins shared similar 3D structures, and a protein–protein interaction network was predicted with known *Arabidopsis* proteins. There were twenty putative ped-miRNAs which were also predicted that belong to nine families targeting thirteen *PeKCS* genes. Gene ontology (GO) and Kyoto Encyclopedia of Genes and Genomes (KEGG) annotation results were highly associated with fatty acid synthase and elongase activity, lipid metabolism, stress responses, and plant-pathogen interaction. The highly enriched transcription factors (TFs) including ERF, MYB, Dof, C2H2, TCP, LBD, NAC, and bHLH were predicted in *PeKCS* genes. qRT-PCR expression analysis revealed that most *PeKCS* genes were highly upregulated in leaves including *PeKCS2, PeKCS4, PeKCS8, PeKCS13*, and *PeKCS9* but not in stem and roots tissues under drought stress conditions compared with controls. Notably, most *PeKCS* genes were upregulated at 9th dpi under *Fusarium kyushuense* biotic stress condition compared to controls. This study provides a basis for further understanding the functions of *KCS* genes, improving wax and VLCFA biosynthesis, and improvement of passion fruit resistance.

## Introduction

Plant growth and development are significantly affected by a variety of biotic and abiotic stresses including drought, salinity, high or low temperature, fungal, bacterial, and viral pathogens. These stress conditions directly affect the crop yield and cause huge economic losses (Raza et al., 2021; Sharif et al., 2021). To adapt and resist these stress conditions, most plants are covered with a hydrophobic protective layer commonly known as cuticle wax and is the first barrier between the environment and plants (Trivedi et al., 2019). Plant cuticle plays an important role in controlling non-stomatal water loss, regulating transpiration, and prevent from mechanical damages caused by fungal, bacterial, insects, ultraviolet (UV) light, and other environmental biotic and abiotic stresses (Lewandowska et al., 2020; Arya et al., 2021; Raza et al., 2022). The structure and composition of cuticle waxes vary between different tissues and between different plants. Different factors affect the biosynthesis and composition of cuticle wax including water, light, temperature, and genotype (Xue et al., 2017). The biosynthesis and transport pathways of cuticle wax are complicated and required the participation of different organelles and enzymes to complete (Fernández et al., 2016; Skalicky et al., 2021). Cuticle waxes are composed of very-long-chain fatty acids (VLCFAs) and their derivatives, such as alkanes, ketones, primary and secondary alcohols, aldehydes, esters, and triterpenes (Zhang et al., 2020). VLCFAs are long-chain fatty acids between C16 and C34 carbons and are the major constituents of sphingolipids, phospholipids, glycerophospholipids, sterol esters, triacylglycerols, and wax esters (Beaudoin et al., 2009). In *Arabidopsis*, sphingolipids containing VLCFAs describe as a secretory pathway for polar plasma membrane protein (Markham et al., 2011).

Biosynthesis of VLCFAs is accomplished in two pathways, including prokaryotic and eukaryotic pathways, involving the *de novo* synthesis of C16 and C18 fatty acids in plastids by fatty acid synthase (FAS) complex (prokaryotic pathway) and elongation of fatty acids from C16 andC18 chains to C26-C34 chains (eukaryotic pathway) via fatty acid elongase (*FAE*) complex in the endoplasmic reticulum (ER) (Bach and Faure, 2010; Xue et al., 2017). Elongation of fatty acids consists of four consecutive reactions in ER including condensation, reduction, dehydration, and secondary reduction and catalyzed by four major enzymes such as β-ketoacyl-CoA synthetase (*KCS*), trans-2,3-enoyl CoA reductase (*ECR*), 3-hydroxacyl-CoA dehydratase (*HCD*) and 3-ketoacyl-CoA reductase (*KCR*) (De Bigault Du Granrut and Cacas, 2016; Kogure et al., 2022). Each of these enzymes utilizes the product of the previous enzyme as a substrate in a cycle starting from the condensation of malonyl-CoA to long-chain acyl-CoA (De Bigault Du Granrut and Cacas, 2016).

β-Ketoacyl-CoA synthase is the key enzyme with distinct substrate specificity that catalyzes the fatty acid elongation and is involved in the synthesis of waxy component precursors during VLCFAs biosynthesis (Wang X. et al., 2017; Yang H. et al., 2021). The first member of *KCS* was identified and functionally characterized in *Arabidopsis* by James et al. (1995), who found *KCS* contributions in the production and storage of VLFACs in developing seeds and finally named as *FAE1*/*KCS18*. Later on, 21 members of the *KCS* gene family were identified and divided into four subfamilies including *KCS1*-like, *FDH*-like, *FAEl*-like, and *CER6* according to the homology of amino acid sequences (Joubès et al., 2008). The KCS proteins have two conserved domains including FAE1/Type III polyketide synthase-like protein domain (FAE1_CUT1_RppA) and 3-Oxoacyl-[acyl-carrier-protein (ACP)] synthase III C-terminal domain (ACP_syn_III_C) (Dai et al., 2021).

Several studies have been reported on the functional characterization of *KCS* genes in plants. It has been reported that five *KCS* genes including *KCS1*, *KCS2*, *CUT1*-CER6/*KCS6*, *KCS9*, and *KCS20* mainly involved in VLCFAs biosynthesis, precursors of cuticular wax and suberin (Lee and Suh, 2013). Beaudoin et al. (2009) reported the involvement of *KCS1* in VLCFAs synthesis, seed triacylglycerols (TAGs), root glycerolipids, and sphingolipids. Todd et al. (1999) reported the involvement of *KCS1* synthase in decarbonylation and acyl-reduction wax synthesis pathways. Overexpression of *KCS2* and *KCS20* increased the wax content in *Arabidopsis* (Lee et al., 2009). *KCS9* was found to be related to tetracosanoic acid as a precursor of epidermal waxes, suberin, sphingolipids, and phospholipids (Kim et al., 2013). *KCS16* was found to be involved in wax biosynthesis and leaf trichomes in *Arabidopsis* (Hegebarth et al., 2017). Yang H. et al. (2021) also reported the involvement of *CsKCS2* and *CsKCS11* in fruit cuticular wax biosynthesis, at the ripening stage in Citrinae species. *KCS* also involved in the cuticular wax synthesis of leaf rice (*Oryza sativa*) (Wang X. et al., 2017). In sunflower (*Helianthus annuus*), *HaKCS1* and *HaKCS2* were expressed in seeds and involved in the elongation of fatty acids from C_18_ to C_20_–C_24_ respectively (González-Mellado et al., 2019). Recently Wang et al. (2022) also reported the involvement of *CsKCS20* in VLCFAs elongation and cuticular wax biosynthesis.

Previous studies also reported the role of *KCS* in environmental stress responses including salt, drought, and biotic stresses. In *Arabidopsis*, *AtKCS*2 and *AtKCS*20 mutants showed higher expression patterns under osmotic stress conditions (Lee et al., 2009). Serra et al. (2009) found that silencing of *StKCS6* in potato (*Solanum tuberosum*) reduced the suberine chain length. Ectopic overexpression of apple (*Malus domestica*) *MdKCS*2 increased the wax contents and resistance to drought conditions in *Arabidopsis* plants (Lian et al., 2021). Yang Z. et al. (2020) reported that ectopic overexpression of grape (*Vitis vinifera*) *VvKCS* in *Arabidopsis* enhanced the tolerance to salt stress at germination and seedling stages. Overexpression of *AhKCS*1 in groundnut (*Arachis hypogaea*) (Lokesh et al., 2019) increased the cuticular waxes and reduced membrane damage under drought stress conditions. Overexpression of *BnKCS*1-1/*BnKCS*1-2 in rapeseed (*Brassica napus*) exhibited increased wax concentration and tolerance to drought under drought conditions (Wang Y. et al., 2020). However, Todd et al. (1999) reported that *AtKCS*1 mutants were less resistant to low humidity conditions at the young stage. Ectopic overexpression of navel orange (*Citrus sinensis*) *CsKCS6* in *Arabidopsis* plants increased the tolerance to drought and salt stress conditions (Guo et al., 2020). Weidenbach et al. (2015) found that the growth rate of barley (*Hordeum vulgare*) *HvKCS*6 mutants was improved underwater limitation conditions. Overexpression of *HvKCS*1 in barley improved the leaf waxes and resistance to barley powdery mildew fungus (Li et al., 2018). In cotton (*Gossypium hirsutum*), *GhKCS13* mutants regulated the cold response by modulating the lipids and oxylipin biosynthesis (Wang Q. et al., 2020).

Transcription factors (TFs) are sequence-specific DNA binding proteins that help recruit the transcriptional machinery to gene promoters, that play an important role in the regulatory network of plant growth and development under normal and stress conditions (Samad et al., 2017). Different TFs have been reported to be involved in the regulation of VLCFAs and wax biosynthesis, including basic leucine zipper (bZIP), myeloblastosis (MYB), wax inducer (WIN), apetala2/ethylene response factor (AP2/ERF), NAC [no apical meristem (NAM), Arabidopsis transcription activation factor (ATAF1/2), and cup-shaped cotyledon (CUC2)], and PUCHI (Ois Roudier et al., 2010; Oshima et al., 2013; Trinh et al., 2019; Guyomarc’h et al., 2021; Han et al., 2022). In addition, the miRNAs (micro-RNAs) are non-coding single-stranded RNAs (approximately 21–30 nucleotides) and play an important role in numerous cellular mechanisms as well as in stress resistance through translational inhibition and/or cleavage of target mRNAs during or after transcription (Zhang et al., 2006; Ohtani, 2017). It has been reported that miRNAs were found to be involved in cuticular wax biosynthesis in *Arabidopsis* such as trans-acting small interfering RNA (tasiRNA) was involved in *CER3* silencing during stem wax production (Lam et al., 2015). Wang Z. et al. (2017) and Su et al. (2021b) conducted a genome-scale study in *Brassica napus* and discovered several miRNAs targeting the fatty acid and lipid metabolism enzymes that could regulate the fatty acid synthesis, lipid transport, and metabolism. Later on, Liu et al. (2019) proposed that the bna-miR165a-5p (*Brassica napus* miRNA) might be involved in the wax biosynthesis process by regulating the putative target BnaA06g40560D gene. Recently, Paul et al. (2020) also identified several miRNAs in passion fruit (*Passiflora edulis*) and predicted their involvement in different molecular and biological process, however, the specific mechanism of all the miRNAs are not clear in passion fruit and need further studies. Genes can be further classified according to their function using Gene Ontology (GO) terms such as biological processes (GO-BP), cellular component (GO-CC), and molecular function (GO-MF). The functional classification of genes can be achieved in species using advanced GO terms (Ashburner et al., 2000; Roncaglia et al., 2013). To understand the biological functions of the gene at the genome level, the Kyoto Encyclopedia of Genes and Genomes (KEGG) is a bioinformatics resource that provides functional information in network pathways (Kanehisa and Goto, 2000; Masoudi-Nejad et al., 2007).

Genome-wide identification of gene families facilitates the study of gene functions by providing a foundation for further functional analysis. Due to the availability of large-scale plant genome sequencing and widespread use of bioinformatics techniques, the *KCS* gene family has been identified in different plant species with diverse members including 21 *KCS* genes identified in *Arabidopsis* (*Arabidopsis thaliana*) (Joubès et al., 2008); 28 in maize (*Zea mays*) (Campbell et al., 2019); 30 in peanut (*Arachis hypogea*) (Huai et al., 2020); 58 in rapeseed (*Brassica napus*) (Xue et al., 2020); eight in grape (*Vitis vinifera*) (Guo et al., 2016); 58 in cotton (*Gossypium hirsutum*), 31 in *G. arboreum* and 33 in *G. raimondii* (Xiao et al., 2016); 33 in barley (*Hordeum vulgare*) (Tong et al., 2021); 96 *KCS* genes were identified in six Citrinae species including13 in *Atalantia buxifolia*, 16 in *Citrus ichangensis*, 21 in *C. medica*, 14 in *C. grandis*, 16 in *C. sinensis* and 16 in *C. clementina* (Yang H. et al., 2021) respectively. Whereas, the *KCS* gene family has not been identified in passion fruit (*Passiflora edulis*). Passion fruit is a perennial evergreen climbing vine and is usually cultivated throughout tropical and subtropical areas of the world. Passion fruit is an economically important fruit plant that is demanded its fresh juice, rich in aroma and distinctive nutritional values (Rizwan et al., 2021). Recently, the passion fruit genome has been sequenced and published (Ma et al., 2021), a comprehensive genome-wide study for the first time to determine the *KCS* gene family in passion fruit was conducted. Moreover, their *in silico* prediction of TFs, subcellular localization, evolutionary relationship *via* phylogenic and syntenic analysis, conserved motifs, gene structures, *cis*-regulatory-elements, prediction of putative miRNA, GO and KEGG annotation have been characterized. The gene expression profiles of several *KCS* genes in different passion fruit tissues under drought stress and *Fusarium kyushuense* fungal stress conditions were analyzed to gain insight into passion fruit *KCS* genes. Our findings will facilitate further functional analysis of *KCS* genes in terms of wax and VLCFA biosynthesis to facilitate genetic improvement of passion fruit against biotic and abiotic stresses.

## Materials and Methods

### Identification of β-Ketoacyl-CoA Synthase Genes in Passion Fruit

To identify and characterize the *KCS* genes in the passion fruit (*Passiflora edulis*) genome, passion fruit proteins, CDS, gff3, genome files were downloaded from the passion fruit genome^1^ (Ma et al., 2021). We performed two methods, which are Basic Local Alignment Search Tool for proteinsT (BLASTp) and Hidden Markov Models (HMMER) search tool, to identify the *KCS* genes in the passion fruit genome. All known KCS family protein sequences of *Arabidopsis thaliana* (*AtKCS*) (Joubès et al., 2008) were downloaded from The *Arabidopsis* Information Resource (TAIR) database^2^, *M. domestica* (Apple) *MdKCS* (Lian et al., 2020) from apple genome^3^, and *A. hypogaea* (Peanut) *AhKCS* (Huai et al., 2020) from peanut genome^4^. The BLASTp was performed in the passion fruit genome based on protein homology alignments with default mode using the known KCS protein sequences *via* the TBTools software package version 1.0986858 (Chen C. et al., 2018). The KCS domains were downloaded from the Pfam database^5^ [PF08392 (FAE1/Type III polyketide synthase-like protein) and PF08541 (3-Oxoacyl-[acyl-carrier protein (ACP)] synthase III C terminal)]. hese domains were used for HMMER in passion fruit genome *via* TBTools with default mode. The results of the two methods were merged and further analyzed by Web CD-Search Tool^6^ and SMART^7^ to confirm the domains in passion fruit KCS protein.

### Physiochemical Properties, Alignment, and Phylogenetic Analyses of *PeKCS* Genes

The *PeKCS* genes’ physical and chemical properties include molecular weight (M.W), amino acid (A.A) length, coding sequence (CDS) length, isoelectric point (pl), instability index (II), aliphatic index (Ai), number of an exon, and intron (E/I) were evaluated by using the ExPASY-Prot^8^. The online CELLO version 0.2.5^9^ program was used to predict the subcellular localization of *PeKCS* genes. The phylogenetic analyses were performed by aligning the full-length KCS protein sequences of *P. edulis* (*PeKCS*), *A. thaliana* (*AtKCS*), *M. domestica* (*MdKCS*), and *A. hypogaea* (*AhKCS*) by Molecular Evolutionary Genetics Analyses (MEGA) software version 10.1.8^10^. The aligned sequences were subjected to neighbor-joining (NJ) tree construction using the MEGA software with 1,000 bootstrap replicates and all other parameters were set to default. The online web tool iTOL^11^ was used to visualize the phylogenetic tree and divided it into four main groups and eight subgroups according to *AtKCS* (Joubès et al., 2008). The *PeKCS* genes distribution and mapping on all 9 assembled and 1 unassembled scaffold passion fruit chromosomes were investigated by using the passion fruit genomic and annotation files *via* Tbtools software (Chen C. et al., 2018). Moreover, the online Sequence Identity and Similarity (SIAS)^12^ tool was used to perform a pairwise sequences identity between passion fruit *KCS* genes.

### *PeKCS* Gene Structures and Motifs Analyses

The *PeKCS* genes structural organizations and chromosome locations were retrieved from passion fruit genomic files. The conserved motifs of PeKCS protein sequences were predicted by using the Multiple Expectation Maximization for Motif Elicitation (MEME) online tools^13^ and the numbers of motifs were set to 10 motifs. The *PeKCS* gene structures and conserved motifs from the passion fruit were visualized by TBTools software.

### *Cis*-Regulatory Element Analysis of *PeKCS* Genes

To predict the putative *cis*-regulatory elements in promoter regions of *PeKCS* genes, the upstream 2,000 bp sequences of all the *PeKCS* genes were extracted from the genomic DNA sequences. Moreover, the promoter sequence of each gene was subjected to the PlantCARE database^14^ and the *cis*-regulatory elements figure was drawn by using TBTools software (Chen C. et al., 2018). Additionally, the numbers, functions, and sequences of putative *cis*-regulatory elements of *PeKCS* genes were summarized and highlighted into plant growth and development, phytohormones responses, and stress-responsive categories.

### Synteny Analysis and Ka/Ks Values Calculation for *PeKCS* Genes

The tandem and segmental or whole-genome duplication (WGD) duplication provides a new understanding of family genes development and genome progression. The homologous *PeKCS* genes with only one intervening gene on the same passion fruit chromosome were considered to be tandem duplicated, while homologs on other chromosomes were considered to be segmental duplications. *PeKCS* duplicated genes were identified and visualized on their physical location on the chromosomes in the passion fruit genome. Gene duplication, synteny analysis, and Ka (non-synonymous)/Ks (synonymous) values calculation of *PeKCS* genes were performed in TBTools. The syntenic relationships of *KCS* genes between *P. edulis*, *A. thaliana*, and *M. domestica* were performed using the MCScanX toolkit, and *KCS* relationships between the target species were visualized by the Advance Circos package in the TBTools software (Chen C. et al., 2018). Furthermore, the multiple collinearity analysis of *KCS* genes in *P. edulis*, *A. thaliana*, *M. domestica*, and *A. hypogaea* whole genomes were also performed by Multiple synteny Plot packages in TBTools software. The Ka), Ks nucleotide substitution rates, and the Ka/Ks ratios of duplicated *PeKCS* genes were annotated using TBTools and divergence time (T, MYA; million years ago) was calculated by the following formula; T = Ks/2x (*x* = 6.38 × 10^–9^) (Ma et al., 2021).

### Protein–Protein Network Interaction, Secondary Structure, and 3D Modeling of *PeKCS*

The online tool STRING version 11^15^ was used to predict and construct the protein–protein interaction network of PeKCS proteins based on *Arabidopsis* homologous proteins. The STRING tool parameters were set as follows; network type-full STRING network; the meaning of network edges-evidence; the minimum required interaction score was set to medium confidence parameter (0.4) and max number of interaction display was no more than 10. The PeKCS proteins’ secondary structures were predicted by SOPMA SECONDARY STRUCTURE PREDICTION METHOD^16^. Three-dimensional modeling (3D) of PeKCS protein was performed using the Phyre2 online tool^17^ with default settings.

### Prediction of Putative Micro-RNAs Targeting *PeKCS* Genes, Gene Ontology and Kyoto Encyclopedia of Genes and Genomes Annotation Analysis

To predict putative miRNA target sites in the *PeKCS* genes, at first, the mature sequences of passion fruit miRNAs were downloaded from published data (Paul et al., 2020). Moreover, the *PeKCS* CDS sequences were submitted to the online psRNATarget Server^18^ with default parameters for prediction of potential miRNAs in *PeKCS* genes. The interaction network between the predicted miRNAs and *PeKCS* target genes was constructed and visualized by Cytoscape software version 3.9^19^ (Kohl et al., 2011). Furthermore, the GO and KEGG annotation analyses were performed by submitting PeKCS protein sequences to the online database eggNOG^20^ and their enrichment analysis were performed in TBTools software (Chen C. et al., 2018).

### Transcription Factor Regulatory Network Analysis

The TFs’ prediction and regulatory network analysis were performed according to Zheng et al. (2020) with slight modifications. The 1000 bp nucleotide sequences from upstream regions of *PeKCS* genes were extracted and were submitted to the Plant Transcriptional Regulatory Map (PTRM)^21^ with *p*-value ≤ 1e^–6^ (Tian et al., 2020) for the prediction of TFs. Cytoscape version 3.9 software (Kohl et al., 2011) was used to construct and visualized the TF regulatory network.

### Expression Analyses of *PeKCS* Genes in Various Tissues

The passion fruit RNA-Seq raw reads were downloaded from National Center for Biotechnology Information (NCBI) Sequence Read Archive (SRA) database^22^ with the following accession numbers (SRP150688) and (PRJNA634206). The peel samples were from yellow (*P. edulis.* Flavicarpa cv Huangjin) and purple (*P. edulis.* Sims cv Tainong) passion fruit cultivars at the ripening stage, while pulp samples were from both cultivars at different fruit developmental stages. The root samples were from purple passion fruit Pingtan-1 cold-tolerant cultivar under two cultivation areas as limestone (L) and sandy dolomite (D) rocky desertification areas. The leave samples were from yellow Huangjinguo (HJG) cold-sensitive and purple Tainong-1 cold-tolerant cultivars under normal temperature (NT) and chilling stress (CS) conditions. The RNA-Seq raw reads were quality controlled and filtered by fastp package (Chen S. et al., 2018) and mapped to the passion fruit reference genome (Ma et al., 2021) with HISAT2 package (Kim and Langmead, 2015) in Ubuntu wsl v 20.04.3^23^. The sequence alignment map (SAM) files were transformed to binary alignment map (BAM) and sorted with Samtools packages (Li et al., 2009). Fragments per kilobase million (FPKM) values were calculated using the limma and edgeR (Law et al., 2016). Due to large differences in FPKM values among different tissues of passion fruit, the FPKM values were transformed to log^2^. Heatmaps were constructed using TB-Tools software (Chen C. et al., 2018) to visualize the expression profiles of *PeKCS* genes in different tissues.

### Plant Material Under Drought and Fungal Stress Conditions

The *KCS* genes play an important role in VLCFA biosynthesis and plant resistance to biotic and abiotic stresses. The *PeKCS* genes expression profiles were studied by applying the drought stress (abiotic) and *Fusarium kyushuense* fungal pathogen (biotic) stressed conditions compared to controls of yellow and purple passion fruit cultivars. To prepare drought-stressed and normal condition samples for *PeKCS* genes expression analysis, seeds of two commercial passion fruit cultivars yellow (*P. edulis.* Flavicarpa cv Huangjin) and purple (*P. edulis.* Sims cv Tainong) were planted in plastic pots filled with peat moss and soil (2:1 ratio). Greenhouse conditions were set as follows; temperature 25 ± 2°C, photoperiod (16-h), and 75% relative humidity. One-month-old passion fruit plants were subjected to dehydration for 10 days and rewatered to study the qRT-PCR (Quantitative real-time polymerase chain reaction) expression profile of *PeKCS* selected genes under drought conditions. Leaves, root, and stem samples of both cultivars with three biological replicates were collected and quickly frozen in liquid nitrogen and were stored at −80°C for further uses. The samples from normally watered plants were taken as controls. In order to prepare the fungal stress samples, fruits of both passion fruit cultivars were collected from a commercial orchard located in Fujian province, China (23°48035.200 N and 117°07008.100 E). Fruits were surface sterilized and infected with the *F. kyushuense* pathogenic fungus following the protocol mentioned in our previous publication (Rizwan et al., 2021). Peels from infected areas were collected after the 9th and 12th days post-inoculation (dpi), while uninfected fruit at 9th and 12th days were used controls.

### RNA Extraction and Quantitative Real-Time Polymerase Chain Reaction Analysis

RNA was extracted from frozen samples using the Tiangen mini-RNA extraction kit (Tiangen, China) according to the manufacturer’s instructions, and RNA was quantified with a Thermo Scientific NanoDrop 2000 UV-Vis Spectrophotometer (Thermo Scientific, United States). One μg of total RNA was used to synthesize the complementary DNA (cDNA) by Takara PrimeScript™ RT Kit with gDNA eraser (TAKARA, China) and cDNAs were diluted to 5x with deionized distilled water. qRT-PCR was performed on a LightCycler^®^ 96 (Roche Applied Science, Penzberg, Germany) in 20 μL reaction mix consisting of 10 μL TB Green master mix solution (TAKARA), 1 μL of each forward and reverse primer (100 μM), 1 μL of cDNA and 7 μL of ddH_2_O. Using *Pe*60s (Munhoz et al., 2015), As an internal reference gene, the qRT-PCR reactions were performed with the conditions as; preincubation at 95°C for 30 s, followed by 45 cycles at 95°C for 10 s, and 60°C for 30 s. Three biological replicates were used for each reaction and the relative gene expression levels were calculated using the 2^–ΔΔCT^ method (Schmittgen and Livak, 2008). The primer used in this study has been provided in Supplementary Table 1.

### Subcellular Localization of *PeKCS2* Gene

The sub-cellular localization of *PeKCS* genes was hypothetically predicted by CELLO v.2.5 and for the validation, the transient expression assay was performed in onion epidermal cells by selecting a *PeKCS*2 candidate gene. To construct a vector for transient expression assays, the pCAMBIA1302 vector with cauliflower mosaic virus 35S (CaMV35S) promoter and green fluorescent protein (GFP) marker in the upstream region of the multiple cloning site (MCS) was used. The *PeKCS* full-length coding DNA sequences (CDS) without stop codon was amplified by PCR using the following primers: 5′-ACGGGGGACTCTTGACCATGGATGGATAGAGAAA GAGA TCTTTTGTCCACG-3′ (*Nco*I) and 5′-TCTCCTTTACTAGTC AGATCTCAGAGTCGCA GGAGGATATCTGT-3′ (*Bgl*II) (underlined are the restriction sites), and cloned into the pMD 19-T vector (Cat# 6013, TAKARA, Shiga, Japan) following the manufactures instructions. The positive clones were confirmed by sequencing (Sangon Biotech Co., Ltd, Shanghai, China), the gene fragment was digested with *Nco*I and *Bgl*II restriction enzymes and ligated into the *Nco*I-*Bgl*II digested pCAMBIA1302 vector using ClonExpress II One Step Cloning Kit (Cat# C112, Vazyme Biotech Co., Ltd., Nanjing, China) and then transferred to *Agrobacterium tumefaciens* EHA105 strain for the following infection. The resulting plasmids were named CaMV35S-*PeKCS2*-GFP and empty vector CaMV35S-GFP as control. These vectors were successfully transformed into onion epidermal cells by agroinfiltration method and GFP expressions were examined by laser scanning confocal microscopy (Olympus, Tokyo, Japan; FV1200) after 24–72 h of agroinfiltration (Xu et al., 2014).

### Statistical Analysis

One-way ANOVA was performed for statistical analyses and Figures were generated by GraphPad Prism version 8.0.1^24^. The comparisons between treated and controlled samples were performed using Student’s *t*-test and were considered statistically significant if *p* < 0.05.

## Results

### Identification and Physiochemical Properties of β-Ketoacyl-CoA Synthase Genes in Passion Fruit

**FIGURE 1:**
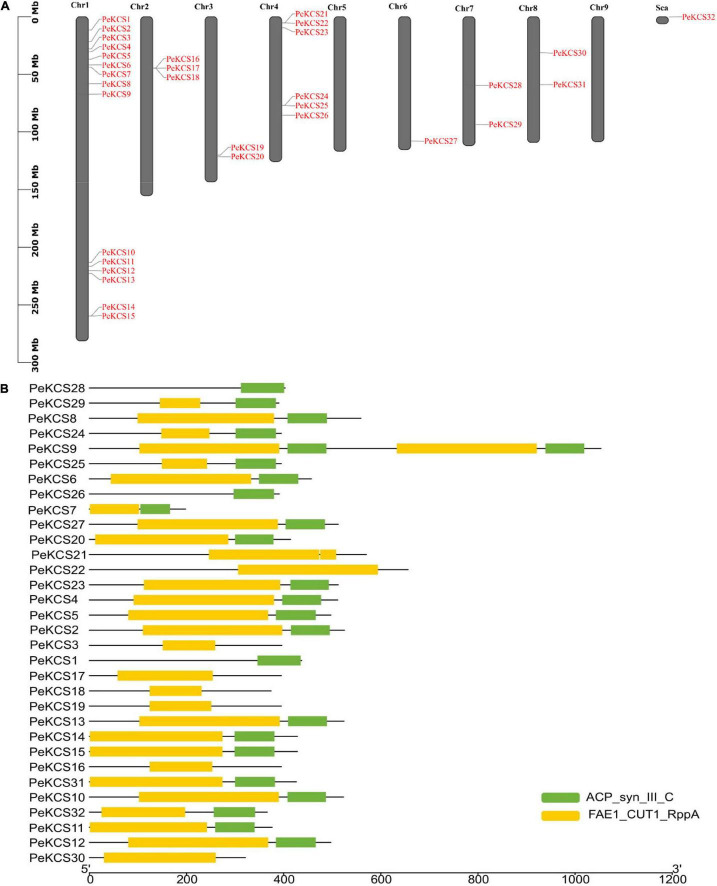
Chromosome location and conserved domains of *PeKCS* in passion fruit. **(A)** Chromosomal location of PeKCS genes, the scale represents the 300 MB chromosomal distance and the PeKCS genes are represented in red color. **(B)** The two conserved domains of PeKCS genes in passion fruit named as FAE1_CUT1_RppA: FAE1 (PF08392); ACP_syn_III_C (PF08541) (readers are referred to the Web version of this article for the understanding of the references to color in this figure legend). Sca-scaffold chromosome.

In this study, after removing redundant, repetitive, and unrecognized sequences from BLASTp and HMMR results, finally, thirty-two *KCS* genes in the passion fruit genome by computational tools were identified. Passion fruit *KCS* genes were named from *PeKCS1* to *PeKCS32* according to their chromosomal positions. Except for chromosomes numbers five and nine, all *PeKCS* genes were unevenly distributed on seven of the nine passion fruit chromosomes and *PeKCS32* was localized on an unassembled chromosome region. The largest number of *PeKCS* genes (14 genes) appeared on chromosome number one, followed by chromosome number four (5 genes), chromosome 2 (3 genes), chromosomes number three, seven, and eight (2 genes each), There is only one *PeKCS* gene on chromosome number six. *PeKCS*32 was found in the scaffold chromosome region, while no *PeKCS* gene was found on chromosomes 5 and 9 (Figure 1A and Table 1). Details of all 32 *PeKCS* genes can be found in Table 1 and the protein sequences have been provided in Supplementary Table 2. CD and SMART search tools for domains verification was used and found that PeKCS proteins contained two domains such as ACP_syn_III_C ((PF08541) 3-Oxoacyl-[acyl-carrier protein (ACP)] synthase III C terminal domain) and FAE1_CUT1_RppA ((PF08392) FAE1/Type III polyketide synthase-like protein domain) (Figure 1B).

**TABLE 1:** Physiochemical properties of *PeKCS* genes.

Gene ID	Gene name	Cr*	Genomic position	CDS (bp)	A.A* (bp)	M.W*	pl*	ll*	Ai*	GR-AVY*	E:I*	SCL*
ZX.01G0003560	*PeKCS1*	1	11711497:11719406−	1311	436	46.68	5.94	31.71	99.36	0.084	9:8	pm
ZX.01G0013920	*PeKCS2*	1	21567509:21569083−	1575	524	58.98	9.17	40.3	91.56	−0.10	1:0	pm
ZX.01G0021050	*PeKCS3*	1	28056974:28058343+	1188	395	43.35	6.85	48.7	88.43	−0.23	2:1	pm
ZX.01G0024040	*PeKCS4*	1	30497870:30500240+	1533	510	57.44	9.38	40.92	95.37	−0.08	2:1	pm
ZX.01G0031680	*PeKCS5*	1	36909208:36910698+	1491	496	56.08	9.38	40.53	100.24	0.02	1:0	pm
ZX.01G0039870	*PeKCS6*	1	42004537:42005907+	1371	456	51.21	8.81	34.12	91.73	−0.06	1:0	pm
ZX.01G0043410	*PeKCS7*	1	43962364:43963095−	594	197	21.96	9.03	39.63	78.22	−0.38	4:3	pm
ZX.01G0060290	*PeKCS8*	1	58285499:58287979−	1677	558	63.18	8.5	44.07	85.11	−0.10	2:1	pm
ZX.01G0068830	*PeKCS9*	1	67253621:67261640−	3159	1052	118.05	9.21	36.2	93.62	−0.08	4:3	pm
ZX.01G0104390	*PeKCS10*	1	213114936:213116972+	1569	522	58.27	8.77	35.34	91.51	−0.07	2:1	pm
ZX.01G0109160	*PeKCS11*	1	216625662:216626789+	1128	375	41.52	8.78	45.41	89.44	−0.15	1:0	pm
ZX.01G0112910	*PeKCS12*	1	220335704:220337194+	1491	496	56.14	9.02	40.08	97.68	0.01	1:0	pm
ZX.01G0116460	*PeKCS13*	1	222578824:222581518+	1572	523	59.33	9.14	38.86	89.29	−0.14	2:1	pm
ZX.01G0132900	*PeKCS14*	1	259611759:259613042−	1284	427	48.22	8.81	35.71	91.57	−0.13	1:0	pm
ZX.01G0133040	*PeKCS15*	1	259664268:259665551+	1284	427	48.17	8.73	35.38	91.33	−0.12	1:0	pm
ZX.02G0008560	*PeKCS16*	2	44526547:44527829+	1185	394	43.01	6.04	40.8	92.59	−0.04	2:1	pm
ZX.02G0008600	*PeKCS17*	2	44614547:44615829+	1185	394	42.98	5.89	42.12	93.32	−0.02	2:1	pm
ZX.02G0008660	*PeKCS18*	2	44697815:44699045+	1122	373	40.87	6.41	41.76	91.02	−0.10	3:2	pm
ZX.03G0023030	*PeKCS19*	3	120077461:120078743−	1185	394	43.01	6.04	40.8	91.85	−0.05	2:1	pm
ZX.03G0023630	*PeKCS20*	3	121353658:121356221+	1242	413	46.55	8.99	34.32	83.58	−0.14	3:2	pm
ZX.04G0004320	*PeKCS21*	4	5525588:5532445+	1710	570	64.17	9.03	40.42	101.11	0.02	6:5	pm
ZX.04G0004390	*PeKCS22*	4	5557716:5565015+	1968	656	73.53	8.91	40.92	95.87	−0.06	5:4	pm
ZX.04G0006280	*PeKCS23*	4	9698999:9701865−	1536	511	57.30	8.83	38.9	100.33	0.04	3:2	pm
ZX.04G0021370	*PeKCS24*	4	76968245:76969643−	1185	394	43.60	6.57	30.01	97.26	−0.08	2:1	pm
ZX.04G0021390	*PeKCS25*	4	77032127:77033535−	1185	394	43.33	6.28	27.47	97.74	−0.06	2:1	pm
ZX.04G0023850	*PeKCS26*	4	85619890:85621186−	1173	390	42.93	6.28	28.46	98.49	−0.08	3:2	pm
ZX.06G0023310	*PeKCS27*	6	107833067:107834602−	1536	511	56.97	9.17	38.3	96.16	−0.01	1:0	pm
ZX.07G0008030	*PeKCS28*	7	59570657:59577025−	1209	402	42.43	6.45	38.95	92.96	0.06	8:7	pm
ZX.07G0016310	*PeKCS29*	7	93550083:93552388−	1170	389	42.81	6.42	38.35	92.21	−0.13	2:1	pm
ZX.08G0007160	*PeKCS30*	8	31405598:31406619−	963	320	36.20	9.14	48.39	81.34	−0.27	4:3	pm
ZX.08G0030480	*PeKCS31*	8	58887421:58888698+	1278	425	47.68	9.3	34.08	90.14	−0.25	1:0	pm
ZX.00000070	*PeKCS32*	Sca	300822:301919+	1098	365	39.07	6.06	37.08	93.1	0.08	1:0	pm

Cr*-chromosome NO; A.A*-amino acid/protein length; M.W*-molecular weight (KDa); pI*-isoelectric point; ll*-instability index; Ai*-aliphatic index; GRAVY*-grand average of hydropathicity; E:I*-No of Exon: Intron; SCL*-Sub-cellular localization; pm*-plasma membrane. Positive (+) and negative (−) signs indicate the presence of a gene on the positive and negative strands of that particular marker at the genome location.

Most of the PeKCS proteins contained both proteins 68% (22 genes), 19% (6 genes) contained only FAE1_CUT1_RppA domains and 9% (3 genes) contained only ACP_syn_III_C domain respectively (Figure 1B). Furthermore, the results of physiochemical properties showed that *PeKCS* genes varied in their properties such as; the protein length varied from 197 bp (PeKCS7) to 1052 bp (PeKCS9), the CDS length ranged from594 bp (*PeKCS*7) to 3159 bp (*PeKCS*9), as well as the protein molecular weight (M.W), ranged from 21.96 KDa (*PeKCS*7) to 118.05 KDa (*PeKCS*9). The isoelectric point (pI) of PeKCS proteins also varied ranged from 5.89 (*PeKCS*17) to 9.38 (*PeKCS*4 and *PeKCS*5), protein instability index (II) varied from 27.47 (*PeKCS*25) to 48.7 (*PeKCS*2) and aliphatic index (Ai) ranged from 81.34 (*PeKCS*30) to 101.11 (*PeKCS*11). The grand average of hydropathicity (GRAVY) ranged from −0.38 (*PeCS7*) to 0.08 (*PeKCS*1). PeKCS protein also varied in number of exons ranged from 1 (PeKCS2, PeKCS5, PeKCS6, PeKCS10, PeKCS11, PeKCS14, PeKCS15, PeKCS27, PeKCS31, and PeKCS32) to 9 (PeKCS1). The proteins subcellular localization prediction showed that all the PeKCS proteins were associated with the plasma membrane (Table 1).

### Multiple-Sequence Alignment and Phylogenetic Analysis of β-Ketoacyl-CoA Synthase Genes

Multiple sequence alignment and evolutionary tree analyses of identified passion fruit *KCS* genes were performed. The multiple sequences analysis between *AtKCS* and *PeKCS* genes exhibited two conserved domains FAE1_CUT1_RppA (Supplementary Figure 1, presented in red box) domain and the ACP_syn_III_C domain (Supplementary Figure 1, presented in the blue box). Both sequences were highly identical and conserved in both regions, indicating the importance of these domains for *KCS* gene functions (Supplementary Figure 1). Furthermore, the N-terminal deletion of the FAE1_CUT1_RppA domain in *PeKCS*7 was pre*cis*e compared to other *PeKCS* genes, suggesting that *PeKCS*7 may have a unique function (Supplementary Figure 1). One hundred and eleven KCS protein sequences from *A. thaliana* (AtKCS), *P. edulis* (PeKCS), *M. domestica* (MdKCS), and *A. hypogaea* (AhKCS) species were used to assess the evolutionary relationship and unrooted phylogenetic tree (All the KCS protein sequences used in the phylogenetic tree have been provided in Supplementary Table 2. The KCS protein sequences were aligned and a neighbor-joining tree was constructed using MEGA software.

**FIGURE 2:**
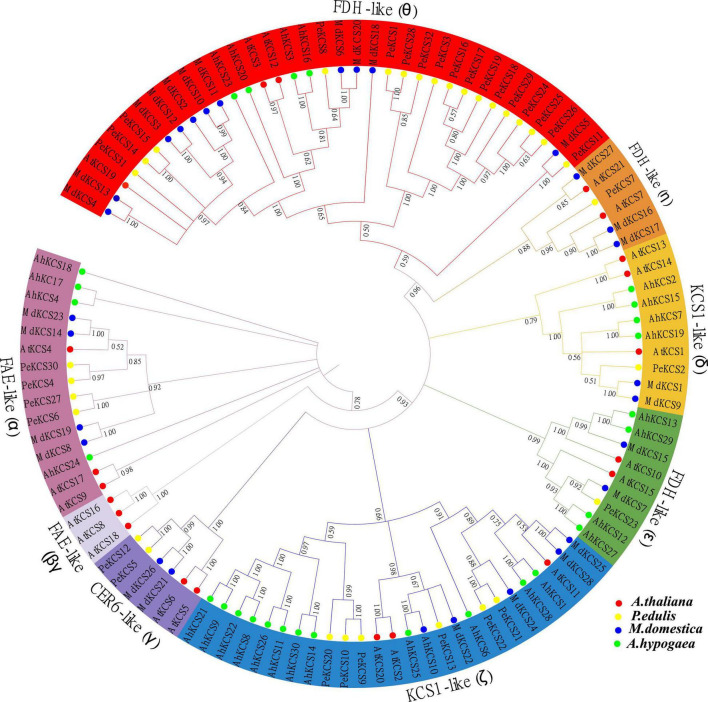
An unrooted neighbor-joining (NJ) phylogenetic tree based on the amino acid sequences alignment among *A. thaliana, P. edulis, M. domestica, and A. hypogaea* KCS sequences with 1000 bootstraps. All the KCS members were divided into 8 groups and presented in different colors. The protein sequences of AtKCS, PeKCS, MdKCS, and AhKCS are represented by red, yellow, blue, and green color circles respectively.

The phylogenetic tree was divided into four main groups (*FAE*-like, *FDH*-like, *KCS1*-like, and*CER6*-like) and eight subgroups including *FAE*-like (α), *FAE*-like (β), *CER6*-like (γ), *KCS1*-like (δ), *KCS1*-like (ζ), *FDH*-like (ε), *FDH*-like (η), and *FDH*-like (θ) (Figure 2) according to *AtKCS* (Joubès et al., 2008). Phylogenetic analysis showed that *FDH*-like (θ) subgroup consisted of 35 *KCS* genes (17 *PeKCS*, 3 *AtKCS*, 11 *MdKCS*, and 4 *AhKCS*) followed by *KCS1*-like (ζ) 26 genes (6 *PeKCS*, 3 *AtKCS*, 5 *MdKCS*, and 12 *AhKCS*), *FAE*-like (α) consists of 15 genes (4 *PeKCS*, 3 *AtKCS*, 4 *MdKCS*, and 4 *AhKCS*), *KCS1*-like (δ) consists of 10 genes (1 *PeKCS*, 3 *AtKCS*, 2 *MdKCS*, and 4 *AhKCS*), FDH-like (ε) consists of 9 genes (1 *PeKCS*, 2 *AtKCS*, 2 *MdKCS*, and 4 *AhKCS*), *FDH*-like (η) consists of 6 genes (1 *PeKCS*, 2 *AtKCS*, and 3 *MdKCS*), *CER6*-like (γ) consists of 6 genes (2 *PeKCS*, 2 *AtKCS*, and 2 *MdKCS*), and *FAE*-like (β) composed of only 3 *Arabidopsis KCS* genes (Figure 2). *FDH*-like (θ) was found to be the largest subgroup consisting of 35 *KCS* genes followed by *KCS1*-like (ζ) 26 genes and *FAE*-like (β) was the smallest subgroup containing only 3 *Arabidopsis KCS* genes. The number of *KCS* genes was similar in *FDH*-like (η) and *CER6*-like (γ). There were also differences in the uneven distribution of *PeKCS* genes between different subgroups, for example, the largest number of *PeKCS* genes (17 genes) was found in the *FDH*-like (θ) subgroup, followed by the *KCS1*-like (ζ) subgroup (6 genes), and only 1 *PeKCS* gene was found in the *KCS1*-like (δ), *FDH*-like (n), *FDH*-like (ε) subgroups and no any *PeKCS* gene was aligned in the *FAE*-like (β) subgroup (Figure 2). The results revealed that *P. edulis* KCS proteins shared greater homology with *M. domestica* (MdKCS) and *A. thaliana* (AtKCS) compared to *A. hypogaea* (AhKCS) (Figure 2). Furthermore, the pair-wise identity between *PeKCS* genes was performed to better understand the evolution and the result showed that the similarity varied from 22.71% (*PeKCS*1/*PeKCS*13) to 99.84% (*PeKCS*16/*PeKCS*17 and *PeKCS*17/*PeKCS*19) respectively (Supplementary Table 3). *PeKCS*17 showed the highest similarity of 99.84% with *PeKCS*16 and *PeKCS*19 genes. Pairwise similarity results were contrasted with phylogenetic tree results, where *PeKCS*16, *PeKCS*17, and *PeKCS*19 genes were grouped in the same *FDH*-like (θ) subgroup (Figure 2 and Supplementary Table 3).

### Gene Structure and Motifs Analyses of *PeKCS* Genes

**FIGURE 3:**
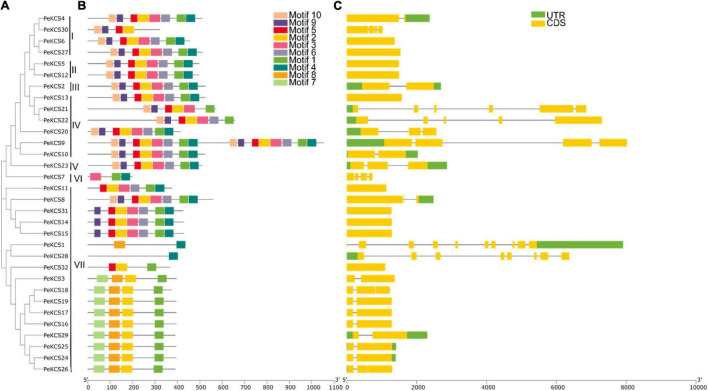
The unrooted phylogenetic tree, conserved motifs and gene structure of *PeKCS* genes. **(A)** The neighbor-joining tree on the left comprised of 32 KCS proteins from passion fruit. **(B)** Conserved motifs were represented *via* boxes and different colors represents different motifs. **(C)** *PeKCS* genes structures, yellow color indicates the exons, the green color shows the untranslated 5′ and 3′-regions, and gray color indicates the introns.

The relationship between 32 *PeKCS* genes was investigated through the construction of a phylogenetic tree using the neighbor-joining (NJ) method and grouped from I-V11 (Figure 3A). The protein sequences of all *PeKCS* genes have been provided in Supplementary Table 2. According to the results, group VII was the largest group with 17 *PeKCS* genes followed by group 4 with 6 genes. The smallest groups were III, V, and VI with only one *PeKCS* gene (Figure 3A). To further understand the development and functions of *PeKCS* family genes, the conserved motifs and exon-intron configuration of *PeKCS* gene structure were analyzed (Figures 3B,C). Moreover, the conserved motifs among 32 *PeKCS* genes were predicted using the online MEME tool. The conserved motifs among PeKCS proteins vary ranged from 1 (*PeKCS*28) to 16 (*PeKCS*9) and overall, 10 conserved motifs were recognized in all PeKCS proteins (Supplementary Table 4). Most of the *PeKCS* genes (12 genes) have 8 motifs followed by 4 motifs (9 genes) and *PeKCS*28 have only one motif. *PeKCS*9 contained a maximum of 16 motifs. The motif distribution among group I-VII was also similar such as groups I, II, III, and V contained 8 conserved motifs except for *PeKCS*30. Furthermore, the 9 members of group VII contained 4 conserved motifs (Figures 3A,B). Genomic structural analysis of exons in 32 *PeKCS* genes revealed that the number of exons identified in *PeKCS* varied from 1 to 9 (Figure 3C). Most *PeKCS* genes (11 genes) consist of 2 exons and 1 exon (10 genes). In addition, *PeKCS*1 contains up to 9 exons (Figure 3C). Group II has only 1 exon and no intron, group 1, III, IV, and V have 1 to 4 introns, group VI have 3 introns and group VII contained 1 to 9 introns except *PeKCS*11, *PeKCS*14, *PeKCS*15, and *PeKCS*32 genes have no introns (Figure 3C). Overall, groups I, II, III, and V showed similar exon-intron patterns whereas groups IV and VII had different exon-intron association patterns (Figure 2C). These results suggest that *PeKCS* genes within a group have remarkably similar gene structures consistent with their phylogenetic relationships (Figure 3). In conclusion, after analyzing the composition of conserved motifs, gene structure, phylogenetic relationships, and group classification, the results indicated that *PeKCS* genes have highly conserved amino acids and genes within a group may have the same functions.

### *Cis*-Regulatory Element Analysis of *PeKCS* Genes

**FIGURE 4:**
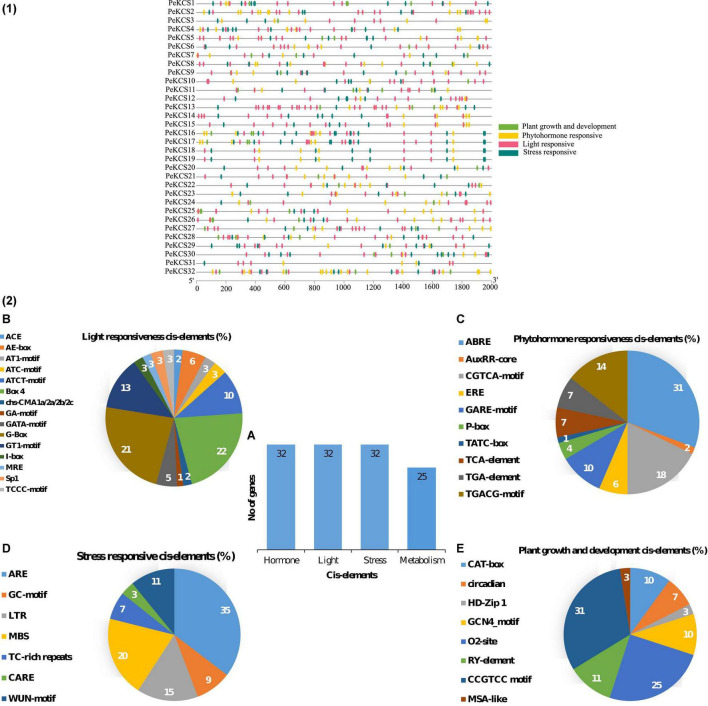
The cis-regulatory element analysis in *PeKCS* genes **(1,2)**. **(A)** The sum number of *PeKCS* genes involved in four categories of cis-elements and the percentage (%) ratio of the numerous cis-elements from each category is presented in pie charts; **(B)** plant growth and development responsive **(C)** phytohormones-responsive, **(D)** light-responsive, and **(E)** stress-responsive. Different colors indicate different cis-acting elements and their ratio present in *PeKCS* genes.

The *cis*-regulatory element analysis of the *PeKCS* genes from the 2000 bp upstream promoter region was conducted to better understand the role of *PeKCS* genes in biotic and abiotic responses (Figure 4 and Supplementary Table 5). Mainly four different categories of *cis*-regulatory elements were found in the *PeKCS* promoter regions including plant growth and development (8 different types of *cis*-elements), phytohormones (10 different types of *cis*-elements), light (15 different types of *cis*-elements), and stress (7 different types of *cis*-elements) responsiveness (Figure 4.1). Overall, a total of 969 *cis*-elements belonging to different categories were identified in 32 *PeKCS* genes, and when comparing between four different categories, the phytohormone-responsive category had the highest number of *cis*-elements at 40% (391/969), followed by light-responsive 37% (351/969), stress-responsive 15% (147/969) and the lowest number of *cis*-elements found in the plant growth and development response category 8.3% (80/969) (Figure 4.2). Furthermore, all the 32 *PeKCS* genes contained the phytohormone, light, and stress-responsive *cis*-elements, whereas only 25 *PeKCS* contained metabolism (plant growth and development) responsive *cis*-elements (Figure 4.2A).

The main four categories were further divided into subcategories based on different *cis*-elements. Light responsiveness category comprised of the 3-AF1 binding site, ACE, AE-box, AT1-motif, ATC-motif, ATCT-motif, TCT-motif, Box 4, chs-CMA1a/2a/2b/2c, GA-motif, GATA-motif, G-Box, GT1-motif, I-box, LAMP-element, MRE, Sp1, and TCCC-motif *cis*-elements (Figure 4.2 and Supplementary Table 5). The highest light-responsive *cis*-elements consisted of Box-4 22% followed by G-Box 21%, while the GA-motif consisted of only 1% *cis*-elements (Figure 4.2B). The phytohormone responsive category further includes ABRE (abs*cis*ic acid-responsive), AuxRR-core (auxin-responsive), ERE (ethylene-responsive), GARE-motif, P-box, TATC-box (gibberellin responsive); TCA-element (salicylic acid-responsive); TGA-element and TGACG-motif (MeJA responsive) *cis*-elements. The highest number of *cis*-elements from the phytohormones category was ABRE 31% followed by CGTCA-motif 18%, while the AuxRR-core consisted of only 1% *cis*-elements (Figure 4.2C and Supplementary Table 5).

The stress response category further included ARE, GC-motif, LTR, MBS, TC-rich repeats, CARE, and WUN-motif *cis*-element. ARE accounted for the highest number 35% followed by MBS with 19%, and CARE with only 3% (Figure 4.2D and Supplementary Table 5). The plant growth and development category further include subcategories of meristem, metabolism, seed related, and circadian. Meristem associated *cis*-elements included CAT-box (10%) and GCN4-motifs (10%). Metabolism-related *cis*-elements included O2-sit (25%), seed and circadian-related *cis*-element included RY-element (11%), and only 3% of MSA-like *cis*-elements were found (Figure 4.2E and Supplementary Table 5). The information on developmental, light, stress, and hormone-related *cis*-elements suggested that the transcriptional profiling of *PeKCS* genes may vary in developmental, hormonal, and stressful contexts and require further investigation. Details on the *cis*-regulatory elements in the passion fruit *KCS* genes have been provided in Supplementary Table 5.

### Synteny Analysis of *PeKCS* Genes

**FIGURE 5:**
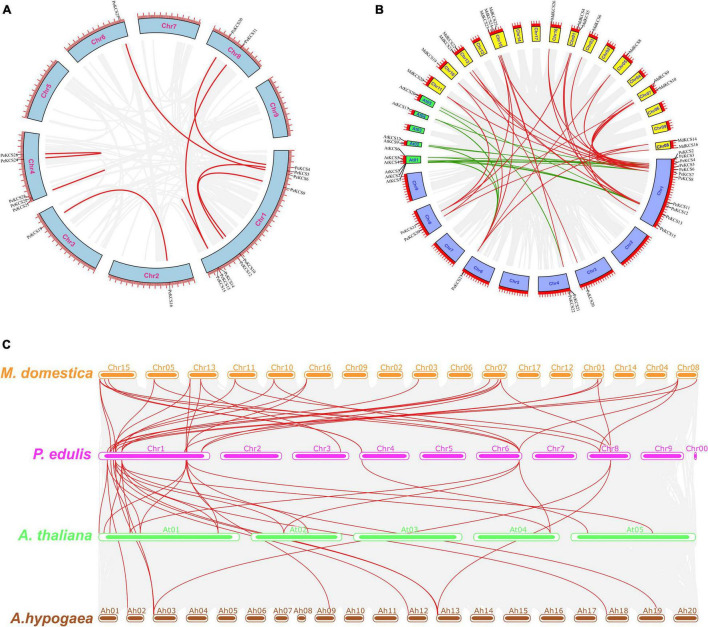
Circos illustrations of the *PeKCS* genes duplication and multiple collinearity analysis of KCS genes between *P. edulis, A. thaliana, M. domestica*, and *A. hypogaea* species. **(A)** Gene duplication of *KCS* genes in *P. edulis*. The background gray lines show all the syntenic blocks in the passion fruit genome, and the red lines show the segmental or tandem duplication link regions among *PeKCS* genes. The approximately location of *PeKCS* genes are labeled with a short gray line outside with gene names. **(B)** Orthologous of *P. edulis KCS* genes with *A. thaliana (AtKCS)* and *M. domestica (MdKCS)* species. Chromosomes of *P. edulis are* represented with Chr1–Chr9, *A. thaliana* with At01-At05 and *M. domestica* with Chr01–Chr17. The background gray lines show all the syntenic blocks genomes of different species, green lines represented the syntenic relationship among *P. edulis* and *A. thaliana* KCS genes, red lines represented the syntenic relationship among *P. edulis* and *M. domestica KCS* genes, **(C)** Multiple collinearity analysis of *KCS* genes between *P. edulis, A. thaliana, M. domestica*, and *A. hypogaea* species. The red lines represent the *P. edulis KCS* genes orthologous in *A. thaliana*, *M. domestica*, and *A. hypogaea* species, the gray lines in the background represent the collinear blocks within *P. edulis* and other species genomes.

Synteny analysis was performed to further understand the evolution and expansion mechanism of the *PeKCS* gene family in the passion fruit genome and the genomes of other species. *PeKCS* gene duplications were assessed based on a tandem or segmental duplications. The results of gene duplication analysis indicate that there were 8 *PeKCS* gene pairs (Figure 5A and Supplementary Table 6), of which 3 gene pairs were segmentally duplicated on chromosomes 1, 2, 3, 6, and 8, whereas 5 gene pairs were tandem duplicated on chromosomes 1 and 4 in passion fruit genome. The duplicated genes belonged to different chromosomes, and chromosome 1 was found to have half of the duplicated genes (8 genes) (Figure 5A and Supplementary Table 6). These results suggest that gene duplication may play an important role in the development of the *PeKCS* gene family and the passion fruit genome. In addition, the Ka/Ks ratios were calculated in duplicated genes to assess the evolutionary rates and selection pressures (Supplementary Table 6). In general, a Ka/Ks ratio greater than 1 indicates that the gene was positively selected, a ratio less than 1 indicates a purifying selection, and a ratio equal to 1 indicates neutral selection. Details of the Ka, Ks values, and Ka/Ks ratios of the duplicated *PeKCS* gene pairs are provided in Supplementary Table 6. Overall, all duplicated *PeKCS* gene pairs showed a Ka/Ks ratio of less than 1, indicating that these genes had gone through purifying selection. Additionally, the divergence time between duplicated genes was measured as a substitution rate of 6.38 × 10^–9^ substitutions per site per year (Ma et al., 2021). The results of the divergence indicated that the duplication process between the tandem and segmental *PeKCS* genes was estimated to be between 0.30 and 27.64 million years ago (Supplementary Table 6). It can be concluded that the evolutionary mechanism of the *PeKCS* gene showed maintenance during the domestication of passion fruit.

Comprehensive synteny analyses of *KCS* genes between *P. edulis*, *A. thaliana*, and *M. domestica* species were performed and 45 *KCS* gene pairs were identified within these three species. Among the 32 *P. edulis KCS* genes, 15 *PeKCS* genes were paired with 26 *KCS* genes in *A. thaliana* (10 *AtKCS*) and *M. domestica* (16 *MdKCS*) (Figure 5B and Supplementary Table 7). Among species, 15 *KCS* gene pairs were found between *P. edulis* (8 *PeKCS*) and *A. thaliana* (10 *AtKCS*) species (Supplementary Table 7). On the other hand, 30 KCS gene pairs were found between *P. edulis* (15 *PeKCS*) and *M. domestica* (16 *MdKCS*) species (Supplementary Table 7 and Figure 5B). Taken together, the *P. edulis* and *M. domestica KCS* genes showed a high degree of synteny compared with *A. thaliana*, suggesting that they may have originated from the same ancestors and performed the same functions, which required further study.

Multicollinearity analysis was performed to reveal robust orthologs of *KCS* genes between *P. edulis A. thaliana*, *M. domestica*, and *A. hypogaea* species (Figure 5C and Supplementary Table 8). The results showed that multiple collinear gene pairs between the four species were inferred as genetic copies with lineage-specific expansion. In general, 58 *P. edulis KCS* genes showed syntenic association with 31 *M. domestica* genes, 16 *A*. *thaliana* genes, and 11 *A. hypogaea* genes. Overall, the maximum collinear genes were found between *P. edulis* and *M. domestica* followed by *P. edulis* and *A. thaliana*, while the fewest were found between *P. edulis* and *A. hypogaea* species. *P. edulis* chromosome 1 shared the maximum collinear genes among *M. domestica* and *A. thaliana* (Figure 5C and Supplementary Table 8), suggesting that the *KCS* genes are conserved and may have the same ancestors besides with duplication or loss of *KCS* genes. Furthermore, many homologs from *A. thaliana*, *M. domestica*, and *A. hypogaea* species maintained a syntenic association with the *P. edulis KCS* gene family suggesting that, in addition to segmental duplications, the whole-genome duplications also play an important role in the evolution of the *PeKCS* gene family.

### Protein–Protein Interaction, Secondary Structure, and 3D Modeling of *PeKCS*

**FIGURE 6:**
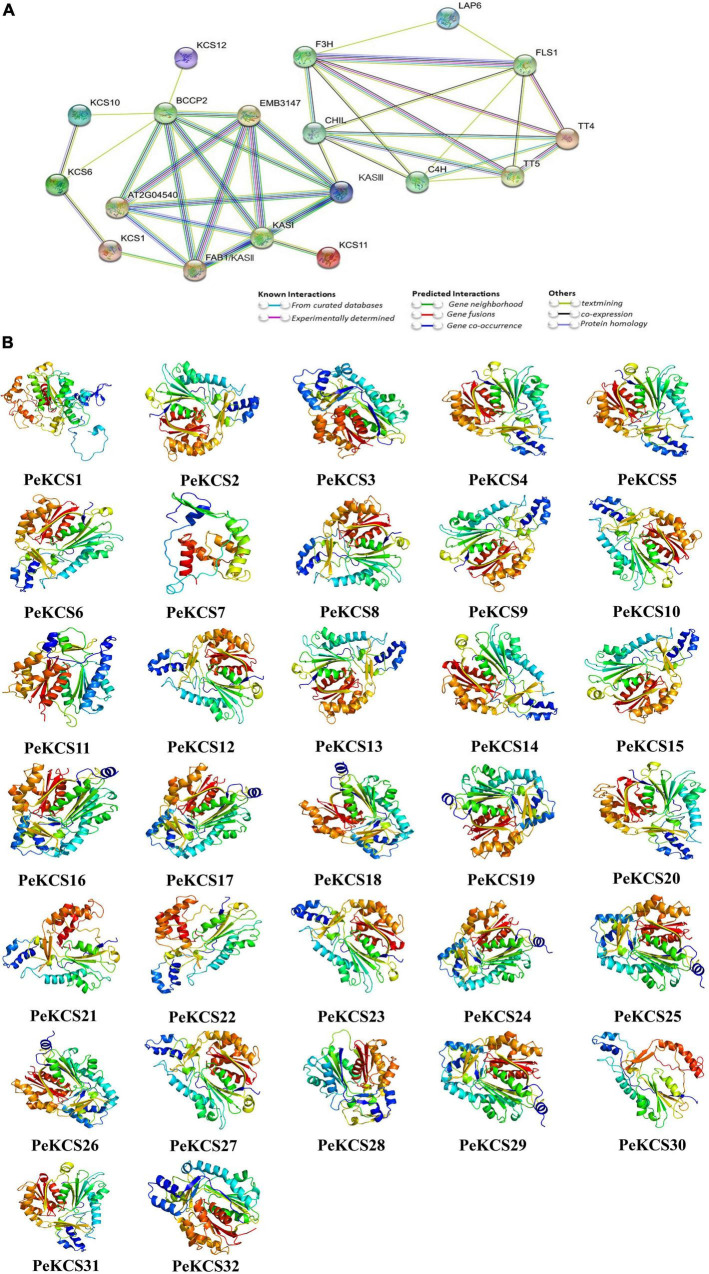
Protein–protein interaction and predicted 3D models of *PeKCS* proteins. **(A)** The network was constructed using online STRING software. The proteins are displayed at network nodes with 3D structure of the proteins within nodes and the lines colors indicate different data sources. The higher the interaction coefficient, the thicker the network lines between proteins and vice versa. **(B)** 3D models were constructed using the online Phyre2 server with default mode.

The protein–protein interaction network analysis of PeKCS proteins based on known *Arabidopsis* proteins was conducted. PeKCS portions having higher homologous similarity with *Arabidopsis* proteins were selected as STRING proteins. Among all, 31 PeKCS proteins were associated with known *Arabidopsis* proteins (Figure 6A and Supplementary Table 9). PeKCS proteins belonging to different groups may have diverse functions in *Arabidopsis*. PeKCS1 and PeKCS28 were homologous with AtKASIII protein and have a strong interaction among AtKASI, AtEMB3147, AT2G04540, and AtBCCP2 proteins. PeKCS2 showed homology with AtKCS1 protein and interacts with AtFAB1/KASI and AtKCS6 proteins. PeKCS5, PeKCS11, and PeKCS12 were homologous with AtKCS6 and showed an interaction with AtKCS6 and AtKCS10 proteins. PeKCS23 shows homology with AtKCS10 and interacts with AtBCCP2 and AtKCS6 proteins. PeKCS8 was homologous with AtKCS12 and interacts with AtBCCP2 protein. PeKCS3 was homologous with AtLAP6 and interacts with AtFLS1 and AtF3H proteins. PeKCS9, PeKCS20, PeKCS21, and PeKCS22 were homologous with AtKCS11 and have a strong interaction with AtKASI protein. PeKCS16, PeKCS17, PeKCS18, PeKCS19, PeKCS24, PeKCS25, PeKCS26, and PeKCS29 were homologous with AtTTP4 and have an interaction among AtFLS1 and AtTT5 proteins. The PeKCS proteins that have strong interaction with *Arabidopsis* proteins might have similar functions as in *Arabidopsis*. The higher the interaction coefficient, the thicker the line between proteins and vice versa (Figure 6A and Supplementary Table 9).

The protein secondary structure generally consists of alpha helices, extended strands, beta turns, and random coils. The PeKCS protein secondary structure analysis was performed and found that in all PeKCS proteins, the alpha helix accounted for the largest percentage of secondary structures 49.6% (PeKCS5) followed by random coils 41.96% (PeKCS1) and extended strands 22.89% (PeKCS28), whereas the beta turns accounts only for 9.2% (PeKCS28). PeKCS28 showed the highest percentage in extended strands and beta turns might have a specific function that needs further studies (Supplementary Table 10). Additionally, the 3D models of all the PeKCS proteins were predicted by using the Phyre2 server with default mode (Figure 6B). The following templates were used for the predicted PeKCS protein models including nineteen PeKCS (PeKCS2, PeKCS4, PeKCS5, PeKCS6, PeKCS7, PeKCS8, PeKCS9, PeKCS10, PeKCS12, PeKCS13, PeKCS14, PeKCS15, PeKCS20, PeKCS21, PeKCS22, PeKCS23, PeKCS27, PeKCS30, and PeKCS31) protein models were predicted with c3vs9F template. Eight PeKCS (PeKCS16, PeKCS17, PeKCS18, PeKCS19, PeKCS24, PeKCS25, PeKCS26, and PeKCS29) protein models were predicted with c1cmlA template. Template c1u0mA in PeKCS32 model, template c2d3mA in PeKCS3 model, template c3gwaA in PeKCS28 model, template c3tsyA in PeKCS11 model and template c4b3yB in PeKCS1 respectively. All PeKCS proteins showed a flexible structure due to the presence of coils (Figure 6B). Our finding suggested that *PeKCS* genes from individual genomes may be ancestrally similar with each other or initially differ maybe stabilized during long-term domestication, leading to changes in protein structure and functions.

### Prediction of Potential Micro-RNAs Targeting *PeKCS* Genes

**FIGURE 7:**
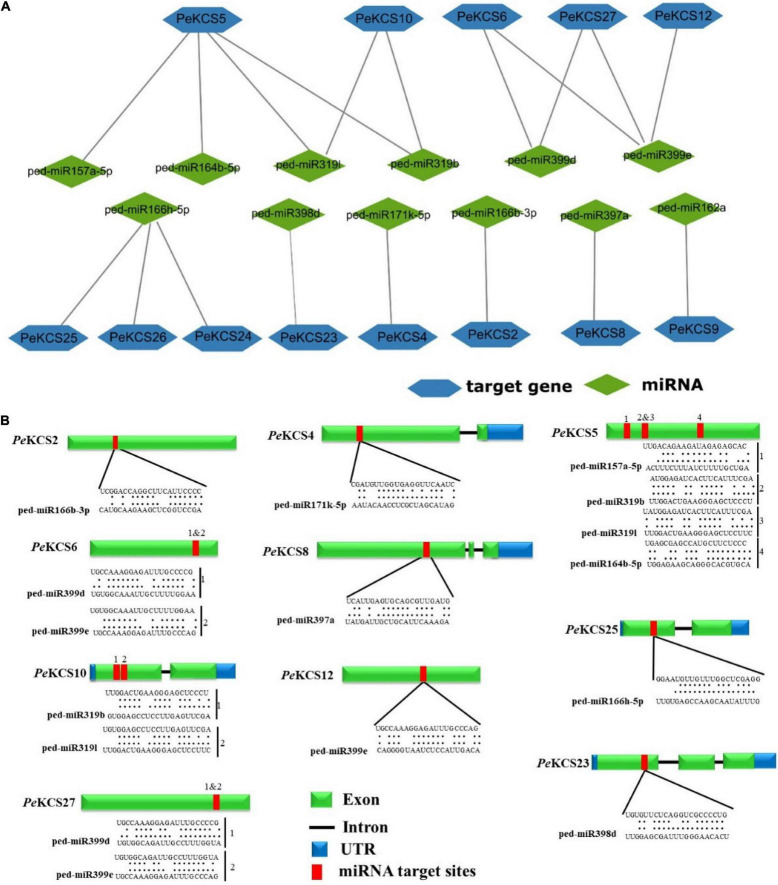
Predicted miRNAs targeting *PeKCS* genes. **(A)** Network illustration of predicted miRNA targeted *PeKCS* genes. Green ellipse shapes represent the predicted miRNAs and bluish hexagon shapes represent targeted *PeKCS* genes. **(B)** The schematic diagram indicates the *PeKCS* targeted by miRNAs and the putative miRNAs sites are indicated by red color, upper sequences are from the gene region and lower sequences are from the miRNAs (Supplementary Table 8 for detailed information of all the predicted miRNAs and targeted *PeKCS* genes).

MicroRNAs are a class of small non-coding regulatory RNAs that control gene expression by directing target mRNA cleavage or translational repression (Sunkar and Zhu, 2004). Over the past few decades, various studies have been reported that miRNAs induce the regulation of stress, plant development, and single transduction (Witkos et al., 2011). Therefore, to better understand the regulatory mechanism of miRNA involved in the regulation of *PeKCS* genes, the identification of putative miRNA in *PeKCS* genes was performed by using the mature sequences of known passion fruit miRNAs as described in the material section. Details of the putative miRNA targeting sites and the *PeKCS* genes have been provided in Supplementary Table 11. Twenty putative ped-miRNAs belonging to 9 different families were identified, targeting the 13 *PeKCS* genes (Figure 7 and Supplementary Table 11). The results show that one member of the ped-miR157 family targeted one gene (*PeKCS*5); one member of the ped-miR162 family targeted one gene (*PeKCS*9); one member of ped-miR164 family targeted one gene (*PeKCS*5); two members of the ped-miR166 family targeted four genes (*PeKCS*2, *PeKCS*24, *PeKCS*25, and *PeKCS*26); one member of ped-miR171 family targeted one gene (*PeKCS*4); two members of ped-miR319 family targeted two genes (*PeKCS*5 and *PeKCS*10); one member of ped-miR397 family targeted one gene (*PeKCS*8); one member of ped-miR398 family targeted one gene (*PeKCS*23) and two members of ped-miR399 family targeted three genes (*PeKCS*12, *PeKCS*26, and *PeKCS*27) (Figure 7 and Supplementary Table 11). Taken together, the results indicated that *PeKCS*5 was most targeted by four putative ped-miRNAs (ped-miR157a-5p, ped-miR164b-5, ped-miR319b, and ped-miR319l) belonging to four distant families, whereas the ped-miR166 family that targeted up to four *PeKCS* genes (*PeKCS*2, *PeKCS*24, *PeKCS*5, and *PeKCS*26) (Figure 7A and Supplementary Table 11). Network and schematic diagram of putative miRNA targeting sites of *PeKCS* genes shown in Figures 7A,B.

### Prediction of Transcription Factor Regulatory Network of *PeKCS* Genes

**FIGURE 8:**
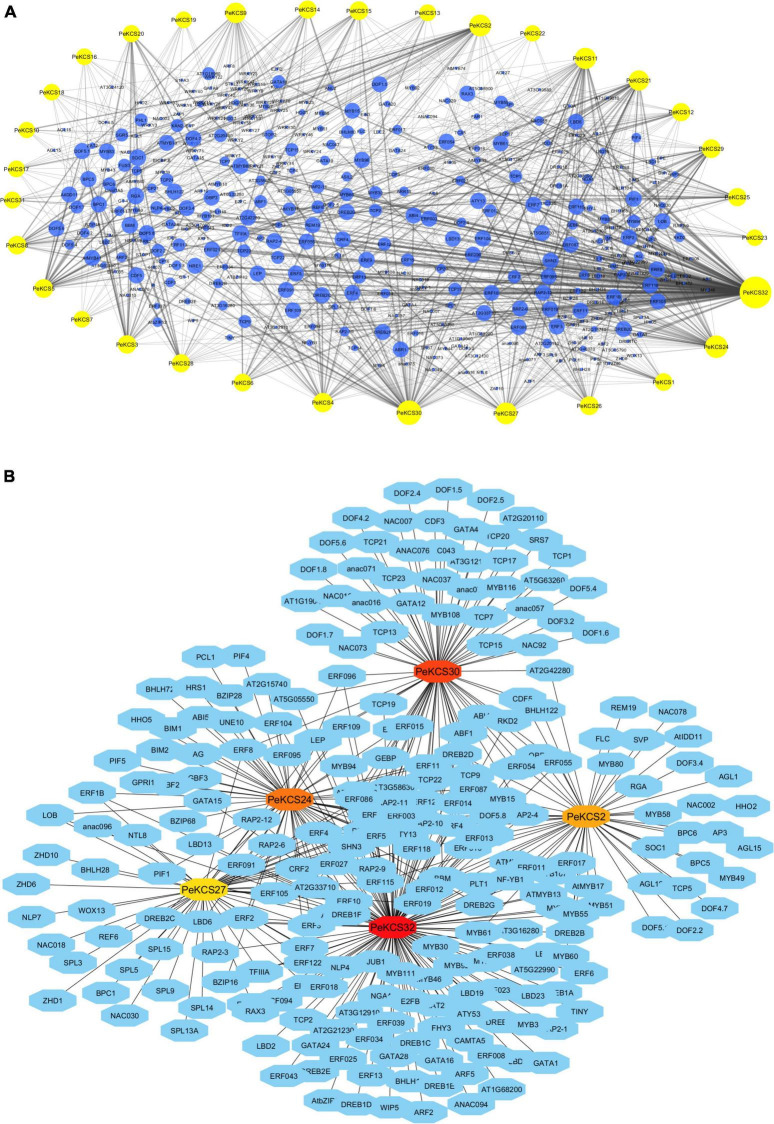
The putative transcription factor regulatory network analysis of *PeKCS* genes. **(A)** Bluish circular nodes represent transcription factors; yellow octagonal nodes represent *PeKCS* genes and node size represents the degree of interaction between nodes based on degree value. **(B)** The top 5 highly enriched and targeted *PeKCS* genes are shown, darker the color shows highly enriched. Transcriptional factors were predicted by PTRM online program and regulatory network was constructed using the Cytoscape 3.9 software.

The potential TFs in the upstream region of (1000bp) of *PeKCS* genes were predicted and constructed the TF regulatory networks (Figure 8). The results showed that, overall, 1701 TFs were identified in 32 *PeKCS* genes belonging to 38 different TF families including ERF, AP2, GRAS, ARF, MYB, Dof, C2H2, TCP, LBD, bHLH, MIKC_MADS, BBR-BPC, and NAC (Figure 8 and Supplementary Table 12). The most abundant TF families were ERF (719 members), MYB (138 members), Dof (102 members), C2H2 (91 members), TCP (75 members), LBD (64 members), and bHLH (61 members) (Supplementary Table 12). Whereas the least abundant TF families were also identified contained only 1 member including WOX, EIL SRS, HSF, BES1, and S1Fa-like (Supplementary Table 12). All 32 *PeKCS* genes were predicted to be targeted by numerous TFs belonging to different families, for example, *PeKCS*32 was the most targeted with 547 TFs followed by *PeKCS*30 with 113, *PeKCS*24 with 76, *PeKCS*2 with 72, and *PeKCS*27 with 64 TFs. Whereas *PeKCS*10 and *PeKCS*22 were minimally targeted genes with only 10 and 9 TFs respectively (Figure 8 and Supplementary Table 12).

Furthermore, a regulatory network of TFs for the top five *PeKCS* genes was constructed based on their degree of interaction, and the results showed that *PeKCS*32 was highly targeted, followed by *PeKCS*30, *PeKCS*24, *PeKCS*2, and *PeKCS*27 (Figure 8B and Supplementary Table 12). *PeKCS* genes were targeted with numerous members of different TF families such as *PeKCS*32 was enriched in ERF (412), LBD (40), and MYB (27) family members, *PeKCS*30 was enriched in ERF (38), TCP (20), and NAC (16) family members. *PeKCS*2 has enriched ERF (17), MYB (14), and MIKC_MADS (13) family members (Figure 8B and Supplementary Table 12). Overall, the ERF family was found to be dominant in all TF families. The TFs regulatory network of all the 32 *PeKCS* genes are shown in Figure 8A, and the networks of the top five highly enriched *PeKCS* genes are shown in Figure 8B, respectively and more details can be found in Supplementary Table 12. The different fatty acids and defense-related TFs belonging to different families were identified in the present study including ERF, AP2, bHLH, WRKY, and MYB. TFs involved in plant growth and development including TCP, bHLH, BBR-BPC, WRKY, LBD, and AP2 were also found in *PeKCS* genes. In addition, phytohormone-related TFs were also identified, including ERF and ARF. Interestingly, ERF, MYB, and AP2 TFs were shown to be universally distributed in most of the *PeKCS* genes (Figure 8 and Supplementary Table 12).

### Gene Ontology and Kyoto Encyclopedia of Genes and Genomes Enrichment Analysis of *PeKCS* Genes

**FIGURE 9:**
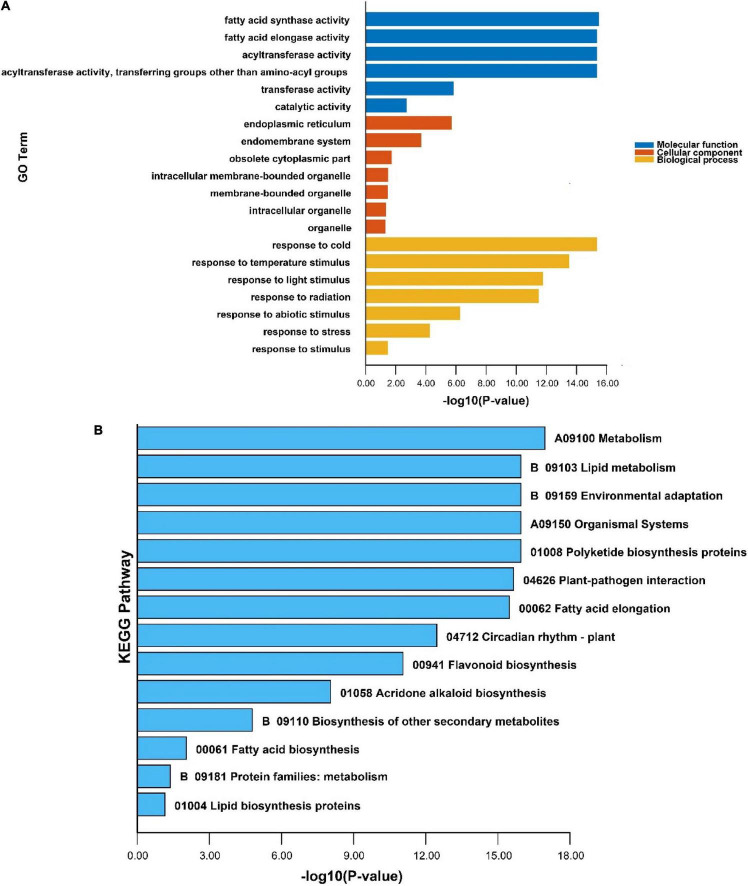
GO and KEGG enrichment analysis of *PeKCS* genes. **(A)** The highly enriched GO terms in *PeKCS* genes. **(B)** The highly enriched KEGG pathways in *PeKCS* genes. Details about GO annotation, enrichment and terms of MF, CC, BP, and KEGG pathways can be found in Supplementary Tables 11, 13a,b.

Gene ontology and KEGG annotation analysis of the *PeKCS* gene was performed to further understand the possible roles of *PeKCS* genes in molecular function (MF), cellular component (CC), and biological process (BP) at the molecular levels. The details of annotation results and numerous significantly enriched terms for MF, CC, and BP have been provided in Supplementary Tables 13a,b. Seventeen terms were identified in 32 *PeKCS* genes belonging to GO-MF class, among which four terms were highly enriched including fatty acid synthase activity (GO:0004312), fatty acid elongase activity (GO:0009922), and acyltransferase activity (GO:0016746, GO:001674747) (Figure 9A and Supplementary Table 13). The GO enrichment analysis showed that GO-CC class exhibited thirty-one terms in 32 *PeKCS* genes, and seven terms were highly enriched including endoplasmic reticulum (GO:0005783), endomembrane system (GO:0012505), obsolete cytoplasmic part (GO:0044444), membrane-bounded organelle (GO:0043227), and organelle membrane (GO:0031090) (Figure 9A and Supplementary Table 13).

For instance, the maximum number of sixty-seven terms were identified in GO-BP class and six terms were highly enriched including response to cold (GO:0009409), response to temperature stimulus (GO:0009266), response to light stimulus (GO:0009416), response to radiation (GO:0009314), response to abiotic stimulus (GO:0009628) and response to stress (GO:0006950) (Figure 9A and Supplementary Table 13). Taken together, the enrichment and annotation results revelated that biological class terms were highly enriched followed by the cellular class in *PeKCS* genes. In addition, KEGG pathway enrichment analysis revealed that 15 pathways were predicted to be involved in different functions among the 32 *PeKCS* genes. Among the predicted KEGG pathways, the highly enriched pathways are presented in Figure 9B, including metabolism (A09100), lipid metabolism (B09103), environmental adaptation (B09159), organismal systems (A09150), polyketide biosynthesis proteins (01008), plant-pathogen interaction (04626), fatty acid elongation (00062) and circadian rhythm (04712) respectively (Figure 9B and Supplementary Table 4). In conclusion, the GO and KEGG enrichment analysis suggests that *PeKCS* genes may play important roles in different biological, molecular, and cellular processes including metabolism, fatty acid biosynthesis, and responses to different biotic and abiotic stresses.

### Subcellular Localization of *PeKCS*2 Gene

**FIGURE 10:**
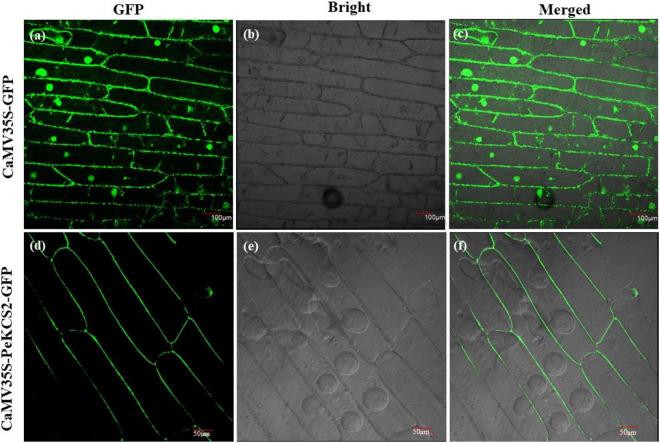
Subcellular localization of GFP-fused *PeKCS*2 protein based on transient expression in onion epidermal cells. **(a–c)** Onion cells transformed with CaMV35S–GFP as control (scale bar = 100 μm). **(d–f)** Onion cells transformed with CaMV35S-*PeKCS*2-GFP (scale bar = 50 μm). **(b,e)** Onion epidermal cells under bright light. **(a–d)** GFP signal under dark field. **(c,f)** represent merged of **(a,b)**, and **(d,e)**, respectively. The transiently expressed in inner epidermis of onion was observed by laser scanning confocal microscopy (Olympus, Japan) after 24 h.

The subcellular localization of all 32 *PeKCS* genes was hypothetically predicted using the online CELLO version 2.5 program. All 32 *PeKCS* genes were expected to localize to the plasma membrane (Table 1). To validate the hypothesized predicted results, *PeKCS*2 was used to perform a transient expression assay in onion epidermal cells. The CaMV35S-*PeKCS*2-GFP fusion construct was transformed into onion epidermal cells by the agroinfiltration method for transient expression assays (Xu et al., 2014). GFP expression was observed and results showed that GFP signals were highly expressed in the plasma membrane (Figures 10d–f). However, the empty vector CaMV35S-GFP was used as a control and the results showed that the GFP was dispersed throughout the cell (Figures 10a–c). The *PeKCS*2 subcellular localization results were consistent with the hypothesized predictions.

### Expression Profiles of *PeKCS* Genes at Different Fruit Developmental Stages

**FIGURE 11:**
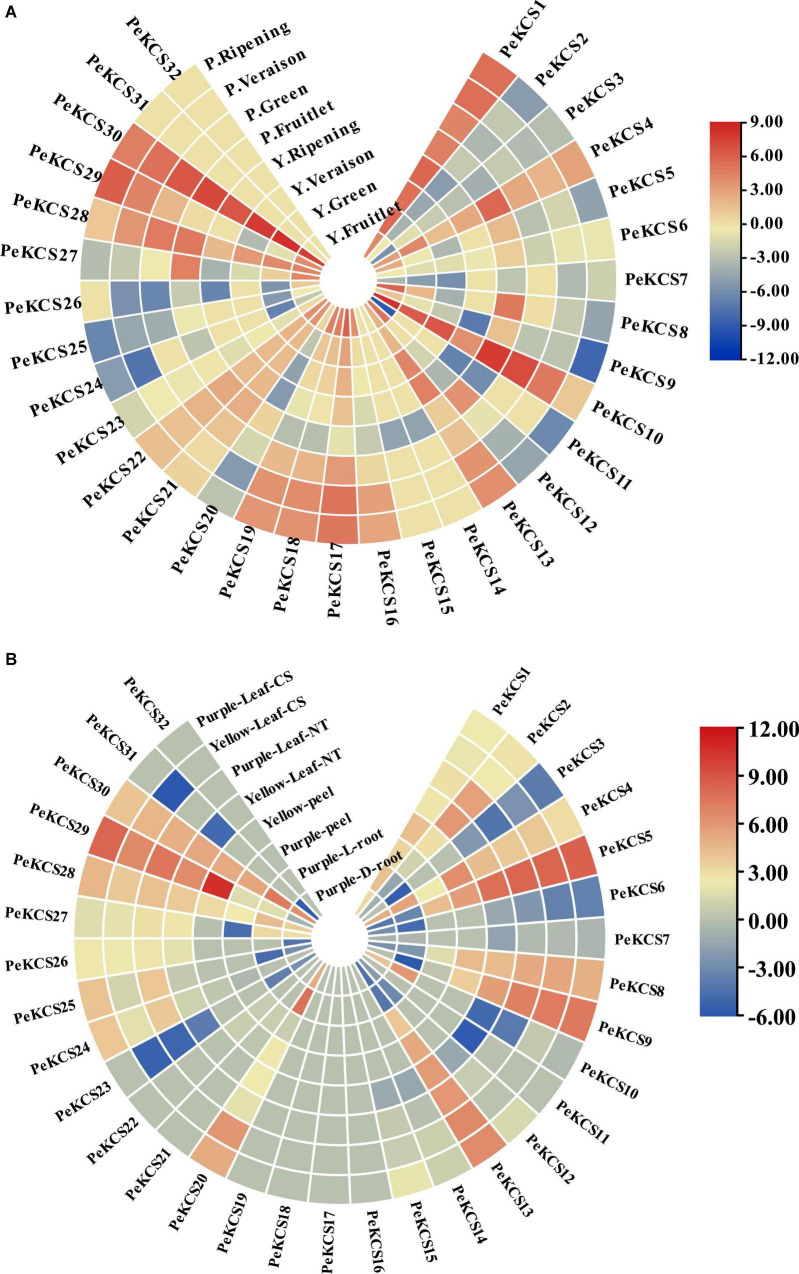
Circular heatmap showing the expression profiles of *PeKCS* genes in pulp, root, peel, and leave tissues of yellow and purple cultivar under different conditions. **(A)** PeKCS expressions in pulp of yellow and purple passion fruit cultivars at different developmental stages. **(B)** *PeKCS* genes expressions in root, peel, and leaves of yellow and purple passion fruit cultivars under different conditions. Y and L represents the yellow (Y) and purple (P) passion fruit cultivars. L and D represents the samples from limestone (L) and sandy dolomite (D) rocky desertification areas. The NT and CS indicate the normal temperature (NT) and chilling stress (CS) conditions. Fragments per kilobase per million (FPKM) values of *PeKCS* genes all tissues were transformed by log2 and heatmap was constructed by TBTools software (the red color shows the highest and the blue color shows lowest expression levels in expression bar).

The expression profiles of all 32 *PeKCS* genes in the pulp tissue of yellow and purple cultivars at different fruit development stages (fruitlet stage, green stage, veraison stage, and ripening stage) based on FPKM values were evaluated. The FPKM expression values have been provided in Supplementary Table 15. The FPKM values were transformed to log^2^ and a circular heatmap was generated by Tbtools software (Figure 11A and Supplementary Table 15). The expression profiles of *PeKCS* genes varied at different fruit developmental stages among both cultivars. Overall, 30 of the 32 *PeKCS* genes were expressed during different fruit developmental stages in both cultivars except *PeKCS*31 and *PeKCS*32. In the yellow cultivar, out of the 30 *PeKCS* expressed genes, 28 (87%), 27 (84%), 24 (75%), and 22 (68%) genes were expressed at fruitlet, green, veraison, and ripening stages. In terms of expression patterns, 14 (50%) genes were highly expressed (FPKM > 10) at the fruitlet stage, 5 (18%) genes at the green stage, 8 (20%) genes at the veraison stage, and 10 (18%) genes at ripening stage. In the comparison of different developmental stages and genes, the expression pattern of *PeKCS*30 (FPKM = 356.7) was highest at the green stage, followed by *PeKCS*10 (FPKM = 324.52) at the fruitlet stage, *PeKCS*30 (FPKM = 233.51) at veraison, and *PeKCS*30 (FPKM = 96.12) at the ripening stage. Only one *PeKCS*13 gene expression was constantly increased during fruitlet (FPKM = 4.71) to ripening stage (FPKM = 23.68), while the expression patterns of five genes (*PeKCS*17, *PeKCS*19, *PeKCS*21, *PeKCS*22, and *PeKCS*28) decreased during fruitlet (FPKM = 69.22, 24.43, 13.16, 14.11, and 20.59) to ripening stages (FPKM = 2.77, 0.6, 3.72, 3.6, and 3.68) (Figure 11A and Supplementary Table 15). Interestingly, three genes had a constant high expression pattern in the four fruit development stages of yellow cultivar, including *PeKCS*1 (FPKM = 17–55.62), *PeKCS*10 (FPKM = 13.73–324.52), and *PeKCS*30 (FPKM = 96.12–356.70) (Figure 11A and Supplementary Table 15).

In the purple cultivar, overall, 30 (93%) *PeKCS* genes were expressed at the fruitlet stage, 25 (78%) at the green stage, 27 (84%) at veraison and ripening stages. Among them, 8 (26%) genes were highly expressed (FPKM > 10) at fruitlet, 5 (20%) at the green, 9 (33%) at veraison, and 7 (26%) at ripening stages. *PeKCS*10 exhibited the highest expression pattern (FPKM = 231.18) at the fruitlet stage, followed by green stage (FPKM = 130.89), *PeKCS*29 (FPKM = 74.68) at the veraison stage, and *PeKCS*1 (FPKM = 43.24) at the ripening stage. The expression patterns of 6 genes (*PeKCS*13, *PeKCS*16, *PeKCS*17, *PeKCS*18, *PeKCS*19, and *PeKCS*19) showed an increased trend from small fruitlet (FPKM = 0.1–1.1) to ripening stage (FPKM = 7.0–74.68), while 3 genes (*PeKCS*10, *PeKCS*27, and *PeKCS*28) decreased expression pattern from fruitlet (FPKM = 23.07–231.18) to ripening stage (FPKM = 0.11–2.3). Remarkably, *PeKCS*1 had a constant high expression pattern in the four fruit development stages of the purple cultivar (FPKM = 25.96–42.53) (Figure 11A and Supplementary Table 15).

In the comparison of *PeKCS* genes between the tested stages, *PeKCS*1 and *PeKCS*30 showed similar higher expressions (FPKM > 10) (Figure 11A and Supplementary Table 15). In the yellow cultivar, the green stage exhibits to have the highest expressions (FPKM = 324.52), whereas in purple the fruitlet stage showed the highest expressions (FPKM = 231.18). Overall, the yellow cultivar had higher expression levels and maximum genes with higher expression patterns compared to the purple cultivar. In addition, the yellow cultivar had decreased an expression pattern (5 genes) from fruitlet to ripening, while purple had increased expression patterns (6 genes) from fruitlet to ripening stages. These findings suggest that *PeKCS* genes may have significant roles in fruit development and ripening stages in both cultivars and further research is still needed (Figure 11A and Supplementary Table 15).

### Expression Pattern of *PeKCS* Genes in Different Passion Fruit Tissues

The expression profiles of *PeKCS* genes in the root, stem, and peel tissues of yellow and purple passion fruit cultivars under different conditions were evaluated in FPKM values, and have been provided in Supplementary Table 16. The expression profiles of *PeKCS* genes in root tissue of the purple, leaf, and peel tissues of yellow cultivars were evaluated in FPKM values and visualized in circular heatmaps by converting to log2 values (Figure 11B and Supplementary Table 16). The expression profiles of *PeKCS* genes varied in different tissues among both cultivars. Overall, out of 32 *PeKCS* genes, 19 (59%) *PeKCS* genes were expressed in purple L and D root tissues, and 15 (46%) *PeKCS* genes were expressed in yellow and purple peel tissues. In contrast, 23 (72%) *PeKCS* genes were expressed in purple leaf NT condition, 25 (78%) in yellow leaf NT, 22 (69%) in purple leaf CS, and 24 (75%) genes were expressed in yellow leaf CS conditions respectively (Figure 11B and Supplementary Table 16). Among the expressed *PeKCS* genes in roots, 6 (31%) and 5 (26%) genes were highly expressed (FPKM > 10) in L and D roots. Roots under the L condition had the highest expression (*PeKCS*20, FPKM = 213.63) compared to roots in the L condition (FPKM = 82.58, *PeKCS*30) (Figure 11B and Supplementary Table 16).

Among the genes expressed in the peel, 8 (53%) and 3 (20%) *PeKCS* genes showed high expressions (FPKM > 10) in purple and yellow peels. Whereas, the purple peel was highly expressed (*PeKCS*29, FPKM = 1692.94) compared to yellow peel (*PeKCS*30, FPKM = 41.97). Under two different conditions (NT and CS), the expression profiles of *PeKCS* genes varied in leaf tissue of both cultivars such as 11 (48%) genes were highly expressed (FPKM > 10) in purple leaf-NT condition, 9 (36%) genes in yellow leaf-NT, 10 (45%) genes in purple leaf-CS and 9 (37%) genes in yellow leaf-CS conditions. Comparing both cultivars under NT and CS conditions, purple leaves under CS had the highest expression (FPKM = 359.34, *PeKCS*5), followed by yellow CS leaves (FPKM = 306.76, *PeKCS*5), purple NT leaves (FPKM = 303.78, *PeKCS*5), and yellow NT leaves (FPKM = 226.04, *PeKCS*5) (Figure 11B and Supplementary Table 16). Taken together, purple had the higher expression in all the tested tissues, whereas, in comparison between tissues, the peel was found to have the highest expression (FPKM = 1692.94, *PeKCS*29) followed by leave tissues (FPKM = 359.34, *PeKCS*5) and root (FPKM = 213.63, *PeKCS*20) (Figure 11B and Supplementary Table 16). Five genes (*PeKCS*16, *PeKCS*17, *PeKCS*18, *PeKCS*19, and *PeKCS*32) were not expressed in all tissues (leave, peel and root) indicating that they might not involve in passion fruit growth and development. These results indicate that *PeKCS* genes have specific expression patterns in all tested tissues and genes with the highest expressions (*PeKCS*5, *PeKCS*29, and *PeKCS*30) might play an important role in specific functions, but further research is required. These genotype-based tissue expression patterns provide ideas for further study of the *PeKCS* gene family in passion fruit.

### Expression Profiles of *PeKCS* Genes Under Drought Stress Condition

**FIGURE 12:**
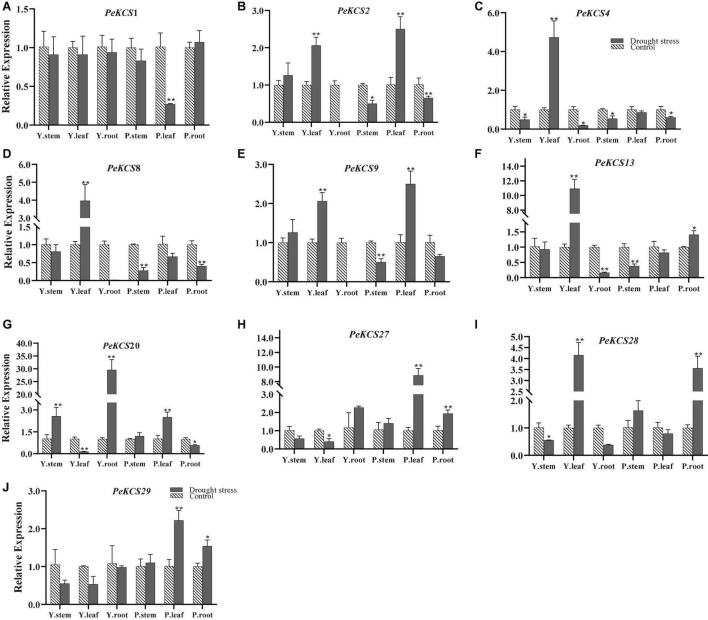
The relative expressions of *PeKCS* genes in stem, root and leave tissues of yellow and purple passion fruit plants under control and drought stress conditions. The relative gene expression levels were calculated using the 2^– Δ^ ^Δ^ *^ct^*. Plants with regular watering were taken as control. Vertical bars represent means ± SD (*n* = 3). The * and ** shows significance at *p* ≤ 0.05 and *p* ≤ 0.01, respectively, among control and drought stress condition according to Students *t*-test. Y, yellow passion fruit; P, purple passion fruit.

The functional characterization of *KCS* genes has not been studied in passion fruit. Gene expression profiles can provide a reflection of gene function. In the current study, the expression profiles of ten candidate *PeKCS* genes were evaluated by qRT-PCR under drought stress conditions (Figure 12). In general, the expression profiles of all candidate *PeKCS* genes showed different expression levels in the stem, leave and root tissues of yellow and purple cultivars under drought conditions. Overall, the results showed that the expression levels of most genes were increased under drought conditions in leave and root tissues compared to controls. The relative expression level of the *PeKCS*20 gene was highest with a more than 29-fold increase in yellow roots under drought conditions (Figure 12G). However, under drought conditions, the relative expression levels of *PeKCS*13 and *PeKCS*27 in yellow and purple leaves increased by more than 10 and 8-folds, respectively (Figures 12F,H). Whereas, relative expressions of *PeKCS*1 decreased in most tissues under drought conditions (Figure 12A). On the contrary, the relative expression levels of *PeKCS*4 and *PeKCS*8 increased by more than 4 and 3-folds in yellow leaves, and all other tissues decreased. Interestingly, *PeKCS*2 showed an increased relative expression in yellow and purple leaves more than 2.5 folds under drought (Figure 12B). Except for *PeKCS*9 and *PeKCS*20, the relative expression levels of most genes decreased in the stem tissue of the yellow cultivar. The relative expression of *PeKCS*28 was increased by more than 4 and 3-folds in yellow leaves and purple roots under drought conditions (Figure 12I). All the yellow tissues showed decreased relative expression levels in *PeKCS*29, while increased in purple tissues under drought conditions (Figure 12J). The leave tissue showed the highest expression consistent with the FPKM expression values (Figure 12 and Supplementary Table 16). Taken together, the results indicated that *PeKCS* genes responded to external drought stress conditions by increasing or decreasing their expression levels in different tissues of the two cultivars, which provided evidence for further functional studies.

### Expression Profiles of *PeKCS* Genes *F. kyushuense* Fungal Biotic Stress Condition

**FIGURE 13:**
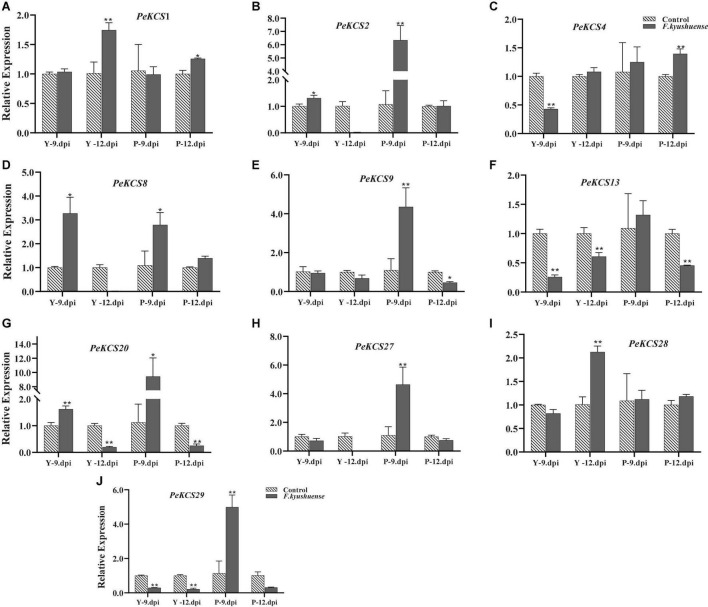
The relative expressions of *PeKCS* genes in peel tissues of yellow and purple passion fruit plants under *F. kyushuense* fungal biotic stress and control conditions. The relative gene expression levels were calculated using the 2^– Δ^ ^Δ^ *^ct^*. Vertical bars represents means ± SD (*n* = 3). The * and ** shows significance at *p* ≤ 0.05 and *p* ≤ 0.01, respectively, among control and biotic stress conditions according to Students *t*-test. Y, yellow passion fruit; P, purple passion fruit; dpi, days post inoculation.

The functional characterization of *KCS* genes against fungal pathogens has not been studied in passion fruit. To gain insight into the potential functional role of *PeKCS* genes at expression levels in passion fruit defense against fungal pathogens as biotic stress, in the current study, the expression profiles of ten candidate *PeKCS* genes were assessed by qRT-PCR under *F. kyushuense* fungal stress (biotic stress) condition in peel tissues of yellow and purple cultivars at 9th and 12th days post-inoculation (dpi) (Figure 13). Overall, all *PeKCS* genes were expressed and showed increased or decreased expression levels under biotic stress conditions in yellow and purple cultivars compared to controls (Figure 13).

All the tested genes showed higher expression levels under biotic stress in purple 9th dpi (P-9.dpi) except *PeKCS*1 (Figure 13A) and *PeKCS28* (Figure 13I) compared to yellow 9th dpi (Y-9.dpi) (Figure 13). Among them, *PeKCS20* (Figure 13G) showed an increased expression level by more than 9-fold, followed by *PeKCS*2 more than 6-fold, *PeKCS*9, *PeKCS*27, and *PeKCS*29 more than 4-fold, *PeKCS*8 more than 2.5-fold, and *PeKCS*4 and *PeKCS*13 more than 1-fold at 9th dpi in purple cultivar (Figure 13). In yellow cultivar at 9th dpi, Only the expression levels of *PeKCS*8, *PeKCS*2, and *PeKCS*20 were found to be increased by 3-fold and more than 1-fold, while the expression level of all other genes decreased at Y-9.dpi (Figure 13). Interestingly, *PeKCS*1 and *PeKCS*28 expression levels were increased by 1.7- and 2.1-folds at 12th dpi in yellow cultivar (Figure 13). Overall, the results showed that *PeKCS* genes expression was increased under biotic stress conditions in purple at 9th dpi compared to controls (Figure 13). Taken together, the results showed that *PeKCS* genes have responded to fungal biotic stress by increasing or decreasing their expression levels at different time points of two cultivars providing evidence for further functional studies.

### Validation of RNA-Seq Expression Data by Quantitative Real-Time Polymerase Chain Reaction

**FIGURE 14:**
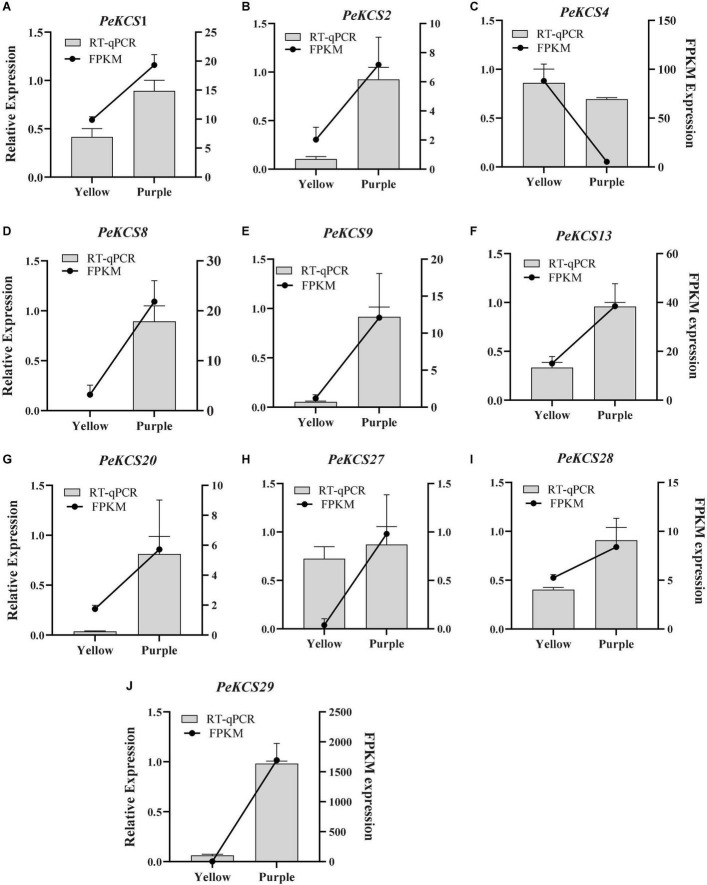
Validation of RNA-seq expression data by qRT-PCR. Histograms show the qPCR results of 10 *PeKCS* genes in peel tissues of yellow and purple cultivars at ripening stage. Black line charts show the FPKM values of *PeKCS* genes on and vertical gray bars show the qPCR results (2^–ΔΔ*ct*^) and represents mean ± SD. The left *Y*-axis represents the relative expression levels by qPCR and the right *Y*-axis indicates the FPKM values of RNA-seq data.

According to RNA-Seq FPKM expression data (Supplementary Tables 15, 16), the *PeKCS*1, *PeKCS*2, *PeKCS*4, *PeKCS*9, *PeKCS*10, *PeKCS*13, *PeKCS*20, *PeKCS*27, *PeKCS*28, and *PeKCS*29 have relative higher FPKM expressions in purple peels compared to yellow. The FPKM expression values were further validated by qRT-PCR analysis using *PeKCS*1, *PeKCS*2, *PeKCS*4, *PeKCS*8, *PeKCS*9, *PeKCS*13, *PeKCS*20, *PeKCS*27, *PeKCS*28, and *PeKCS*29 genes in yellow and purple passion fruit peel tissues (The primer details can be found in Supplementary Table 1). After normalization with Pe60s reference genes, all the tested *PeKCS* genes showed a trend line consistent with the RNA-Seq expression values (Figure 14). These results revealed that RNA-Seq expression values provided an appropriate expression result for all the tested tissues among both passion fruit cultivars.

## Discussion

The plant cuticle wax is the first barrier between the environment and plants to protect them from biotic and abiotic stresses (Trivedi et al., 2019). Plant cuticle consists of a combination of VLCFAs and their derivatives (Yeats and Rose, 2013). VLCFAs are fatty acids consisting of C_16_ to C_40_ carbons that play important roles in plant development and are essential for the synthesis of seed storage triacylglycerols, epicuticular waxes, and sphingolipids (Roudier et al., 2010). Elongation of C_16_ and C_18_ fatty acids to VLCFAs (C_20_ to C_40_) consists of four enzymes in the endoplasmic reticulum (ER) such as *KCS*, *KCR*, *ECR*, and *HCD* (Raffaele et al., 2009). Among them, *KCS* is known to be the key enzyme for VLCFAs biosynthesis (Wang X. et al., 2017; Yang H. et al., 2021). Due to the important roles of *KCS* in plant cuticle synthesis and other biological processes, the genome-wide identification of the *KCS* gene family has been reported in various plant species including *Arabidopsis thaliana* (Joubès et al., 2008), *Zea mays* (Campbell et al., 2019), *Arachis hypogea* (Huai et al., 2020), *Brassica napus* (Xue et al., 2020), *Vitis vinifera* (Guo et al., 2016), *Gossypium hirsutum*, *G. arboreum*, *G. raimondii* (Xiao et al., 2016), *Hordeum vulgare* (Tong et al., 2021), *Atalantia buxifolia*, *Citrus ichangensis*, *C. medica, C. grandis*, *C. Sinensis*, and *C. clementina* (Yang H. et al., 2021) ranging from 16 to 58 *KCS* members. In addition, the *KCS* family members have been functionally characterized in *Arabidopsis* (Lee et al., 2009; Kim et al., 2013; Hegebarth et al., 2017), *C. sinensis* (Yang H. et al., 2021; Wang et al., 2022), *O*. *sativa* (Wang X. et al., 2017), *H. annuus* (González-Mellado et al., 2019), *S. tuberosum* (Serra et al., 2009), *M. domestica* (Lian et al., 2021), *V. vinifera* (Yang Z. et al., 2020), and *A. hypogaea* (Lokesh et al., 2019). However, information about the *KCS* gene family in passion fruit was unknown and the availability of the passion fruit genome (Ma et al., 2021) made it possible to study on genomic levels. Therefore, identification and analyzing the physicochemical properties, structure, and function of the passion fruit *KCS* gene at the genome level could provide a theoretical basis for functional characterization to improve the stress resistance and genetic improvement of passion fruit.

In the current study, a total of 32 *PeKCS* genes were identified in the passion fruit genome by BLASTp and HMMER search basis (Table 1 and Figure 1A). The number of identified *KCS* genes in the passion fruit genome was relatively higher (32 *PeKCS* genes) compared with *Arabidopsis* (21 *AtKCS* genes) (Joubès et al., 2008), which may be due to large differences in genome size and a high number of repeated sequences. Our results are inconsistent with rapeseed (58 *BnKCS* genes) and barley (33 *HvKCS*) (Tong et al., 2021), in which they also identified a higher number of KCS genes (Xue et al., 2020) compared to *AtKCS*. The two domains (FAE1_CUT1_RppA and ACP_syn_III_C) were conserved in PeKCS proteins such as 22 PeKCS proteins have both domains, 6 PeKCS proteins have only FAE1_CUT1_RppA domain and 3 PeKCS proteins contained only ACP_syn_III_C domain (Figure 1B). Abbadi et al. (2000) also reported that the ACP_syn_III_C domain is important for initiating the fatty acid synthase chain reactions in plants and bacteria. KCS protein members with the FAE1_CUT1_RppA domain are described as 3-ketoacyl-CoA synthases and contain the active motifs involved in substrate binding and might be related to the substrate specificity of the *KCS* enzyme (Sagar et al., 2015). These reports support our results, that genes with ACP_syn_III_C and FAE1_CUT1_RppA domains identified in this study belong to the *KCS* gene family. Later on, multiple sequences alignment analysis between *AtKCS* and *PeKCS* genes was performed and found the two conserved domains regions indicating the importance of these domains to *KCS* gene functions (Supplementary Figure 1). Similar results were found by Lian et al. (2021) in *M. domestica* and Lokesh et al. (2019) in *A. hypogaea*.

The phylogenetic tree was constructed among KCS protein sequences of AtKCS, PeKCS, MdKCS, and AhKCS species (Figure 3) and exhibited into four subfamilies and eight clades including *FAE*-like (α), *FAE*-like (β), *CER6*-like (γ), *KCS1*-like (δ), *KCS1*-like (ζ), *FDH*-like (ε), *FDH*-like (η), and *FDH*-like (θ), which are consistent with previously published reports (Joubès et al., 2008; Lokesh et al., 2019; Lian et al., 2021). However, all the 32 *PeKCS* genes were grouped into seven clades and no *PeKCS* gene was found in FAE-like (β) clade (Figure 3). *PeKCS* genes in the KCS1-like (δ) clade were closely related to *AtKCS*1, which were involved in decarbonylation and acyl-reduction wax synthesis pathways (Todd et al., 1999). *PeKCS* genes in *KCS1*-like (ζ) clade were grouped with *AtKCS*2 and *AtKCS*20, which were involved in wax biosynthesis and tolerant to osmotic stress conditions (Lee et al., 2009). *PeKCS* genes in *FAE*-like (α) clade were grouped with *AtKCS*9 and *AtKCS*4, which were involved in VLCFAs, waxes, suberin, sphingolipids, and phospholipids (Kim et al., 2013, 2021). *FAE*-like (β) clade contained the *AtKCS*16, which were involved in the elongation of C_34_-C_36_ and C_38_ VLCFA required for wax biosynthesis and leaf trichomes in *Arabidopsis* (Hegebarth et al., 2017). *PeKCS* genes in *FDH*-like (ε) clade were grouped with *AtKCS*10, which were involved in organ development (Yang T. et al., 2021). These findings suggested that genes present in the same clade might perform similar functions (Liu et al., 2018).

Differences in protein functions can be described by predicting conserved motifs during the development of different gene families (Wong et al., 2015). Furthermore, the conserved motifs in *PeKCS* genes were evaluated and found a diverse motif pattern ranging from 1–16 motifs, whereas, 10 conserved motifs were recognized in all 32 *PeKCS* genes. The motif distribution contrasts with different groups of the evolutionary tree, suggesting that these conserved motifs may be functionally distinct (Figure 2B). Our results are similar to Lian et al. (2021) who also reported a different distribution of motifs and identified 15 conserved motifs in *MdKCS*s. The existence of exons and introns in gene structure (especially intron) provides an important source for determining gene family variation, function, and expressions (Xu et al., 2012). Additionally, the intron number and length negatively correlated with the expression level of corresponding genes (Eghbalnia et al., 2020; Su et al., 2021a). In the current study, a quite different number of introns among 32 *PeKCS* genes ranging from 0 to9 introns were identified and group II was found with having no introns, whereas groups 1, III, IV, and V have 1 to 4 introns, group VI has 3 introns and group VII contained 1 to 9 introns except *PeKCS*11. *PeKCS*14, *PeKCS*15 and *PeKCS*32 genes have no introns (Figure 2C). Similar results for exon/intron were also reported by Lian et al. (2021) and Tong et al. (2021), where different intron and exon patterns were found in the *KCS* genes. These findings suggest that the *PeKCS* gene ancestor may have undergone several rounds of intron loss and gain during development (Frugoli et al., 1998).

The *cis*-regulatory element analysis in *PeKCS* genes was performed and found numerous *cis*-elements that were involved in different plant developmental and stress responses. The *cis*-elements including ABRE, G-Box, Box 4, CGTCA-motif, TGACG-motif, GARE-motif, and ARE were abundant (Figure 4), indicating that *KCS* genes may play an important role in plant hormone response and stresses (Porto et al., 2014). *PeKCS cis*-element results were consistent with the qRT-PCR expression analysis, as genes containing the larger number of stress-related *cis*-elements were highly upregulated under stress conditions, including *PeKSC2*, *PeKCS9*, *PeKCS13*, and *PeKCS28* genes were highly upregulated in drought stress condition (Figure 12), while *PeKCS2*, *PeKCS8*, *PeKCS9*, and *PeKCS27* genes were highly upregulated under F. kyushuense fungal biotic stress condition (Figure 13) Kim et al. (2011) reported that the promoter ABRE sequence was involved in the expression of the DREB2A gene under osmotic stress conditions. Sibéril et al. (2001) proposed that G-BOX might involve in the transduction pathway that regulates the gene expression in the nucleus. These results suggest that genes containing the above-mentioned *cis*-elements might involve in different plant developmental and stress responses as reported earlier (Wingender et al., 1990; Wang et al., 2011; Liu et al., 2016; Xiong et al., 2020). Different species such as *Arabidopsis*, peanut, apple, grape, rapeseed, cotton, sunflower have different numbers of chromosomes and *KCS* genes, suggesting that they might have gone through lineage-specific genome duplication, which is a prominent feature of plant genomes that can lead to evolution and functional novelty from existing genes (Flagel and Wendel, 2009). Guo et al. (2016) reported that the WGD/segmental duplication plays a major role in the expansion of plant *KCS* genes. WGD/segmental and tandem duplications may lead to a sudden increase in the size of the genome and the entire gene sets by generating two gene copies (Van de Peer et al., 2009).

Synteny analysis was performed and calculated *PeKCS* genes divergence rate and found less than 1 Ka/Ks values, suggesting that *PeKCS* genes have gone through purifying selection and the duplication process among tandem and segmental *PeKCS* genes was estimated to be 0.30 to 27.64 mya (Supplementary Table 6), which is inconsistent with Xia et al. (2021), who reported that, the passion fruit experienced a two WGD between 12 mya and 65 mya. *PeKCS* gene duplication analysis showed 3 segmental and 5 tandems duplicated *PeKCS* genes within passion fruit genome (Figure 5A and Supplementary Table 6), correspondence with *Arabidopsis KCS* duplication by Joubès et al. (2008). Furthermore, comprehensive *KCS* gene duplication showed a higher number of KCS duplications were between *PeKCS* and *MdKCS* compared to *AtKCS* (Figure 5B and Supplementary Table 7). The multiple collinearity analysis between *P. edulis A. thaliana*, *M. domestica*, and *A. hypogaea* species (Figure 5C and Supplementary Table 8) also revealed the higher number of orthologous between *P.* edulis and *M. domestica*. Similar results were reported by Santos et al. (2014) that compared to the dicot genomes the number of repetitive elements between *P. edulis* and *M.domestica* genomes was 42.4%. These findings indicate that these orthologous might have the same ancestors and retain corresponding functions.

Protein–protein interaction networks for specific gene families provide evidence of the relationship between known family members (Piya et al., 2014). In the current study, 31 PeKCS proteins showed homology and interaction with known *Arabidopsis* proteins (Figure 6A and Supplementary Table 9). The results showed that PeKCS proteins had homology and interaction with AtKASI, AtLAP, AtKASIII, AtKCS, AtBCCP, AtTTP4. Our results are consistent with published reports suggesting that PeKCS have similar functions, such as Chen et al. (2009) revealed that BCCP (Biotin carboxyl carrier protein) was involved in the fatty acid biosynthesis pathway. Wu and Xue (2010) reported that *KAS* (β-Ketoacyl-acyl carrier protein synthase) catalyzes the elongation of *de novo* fatty acid synthesis and plays a role in chloroplast division and embryo development. Su et al. (2018) stated that *KASII* (Fatty acid biosynthesis I (FAB1)/3-oxoacyl-acyl-carrier-protein synthase II) catalyzes the fatty acid synthesis by conferring resistance to low temperatures by maintaining the integrity of chloroplast membranes. *KCS* is the key enzyme involved in VLCFA biosynthesis and cuticle wax biosynthesis pathways (Lee et al., 2009; Wang X. et al., 2017). Dobritsa et al. (2010) stated that LAP5/LAP6 (Less adhesive pollen) are multifunctional enzymes that might play role in pollen fatty acids and exine phenolic. Xuan et al. (2018) stated that TT4/TT5 (Transparent Testa) encodes chalcone synthase (CHS), a key enzyme involved in the biosynthesis of flavonoids and auxin transport. The proteins’ secondary structures are the physical arrangement of amino acid sequences and are highly conserved between homologous proteins. The amino acid order directly affects the protein folding, 3D structure, and functions (Ridout et al., 2010).

Protein secondary structure analysis revealed that the alpha helix accounts for the largest percentage of secondary structures 49.6%, followed by random coils 41.96%, and extended strands 22.89% (Supplementary Table 10). Similar results have been reported by Lian et al. (2020) in the apple *KCS* gene family. In addition, 3D models of all the PeKCS proteins showed similar structures among them (Figure 6B), indicating that the identified PeKCS proteins were conserved and consistent with the domains, motif, and gene structure analysis (Lian et al., 2020). Recently, different miRNAs have been identified in numerous species including *Brassica napus* (Su et al., 2021b; Wen et al., 2021), maize (*Zea mays*) (Aravind et al., 2017), in cowpea (*Vigna unguiculata*) (Barrera-Figueroa et al., 2011), soybean (*Glycine max*) (Song et al., 2011), *Arachis hypogaea* (Zhao et al., 2015) and passion fruit (Paul et al., 2020), and involved in different metabolism, development, and environmental stresses. In this study, twenty putative ped-miRNAs belonging to nine different families were identified and targeted the 13 *PeKCS* genes (Figure 7 and Supplementary Table 11). These miRNAs included ped-miR157, ped-miR162, ped-miR164, ped-miR166, ped-miR171, ped-miR319, ped-miR397, ped-miR398 and ped-miR399, respectively. Ped-miR166 family targeted up to four *PeKCS* genes and miR166 has been reported to be involved in drought stress in maize (Aravind et al., 2017), cowpea (Barrera-Figueroa et al., 2011); soybean (*Glycine max*) seed development (Song et al., 2011); peanut disease resistant (Zhao et al., 2015); plant growth, development and stress response in apple (Varkonyi-Gasic et al., 2010). miR171 has been reported to be involved in the development, metabolism, photosynthesis in grapevine (*Vitis vinifera*) (Han et al., 2014); coffee (*Coffea Arabica*) (Chaves et al., 2015); tea (*Camellia sinensis*) stress response (Zhang et al., 2014); passion fruit development and defense (Paul et al., 2020). miR399 involved in drought stress response in sugarcane (*Saccharum officinarum*) (Zanca et al., 2010); phosphate homeostasis, signaling, transport in apple (Pant et al., 2008) and sorghum (*Sorghum bicolor*) phosphate deficiency (Katiyar et al., 2012a). These findings suggest that these ped-miRNAs may play key roles in multiple developments and stress processes by altering the transcriptional level of *KCS* genes in passion fruit. The functions and expression levels of these predicted miRNAs and their target genes need to be further examined in the passion fruit.

Plant TFs have been reported to be involved in the regulation of fatty acids and wax biosynthesis under different conditions (Hao et al., 2017). The TFs in the promoter regions of 32 *PeKCS* genes were predicted and proposed a regulatory network interacting with *PeKCS* genes (Figure 8 and Supplementary Table 12). The most abundant TF families were ERF, MYB, Dof, C2H2, TCP, LBD, NAC, and bHLH (Supplementary Table 12). Go et al. (2014) reported that wax biosynthesis in *Arabidopsis* was negatively regulated under AP2/ERF-type TFs. Overexpression of AP2/ERF-type TFs increased the wax load and drought resistance in *Arabidopsis* and tobacco (*Nicotiana tabacum*) (Yang S. U. et al., 2020). MYB TFs regulate VLCFAs biosynthesis, plant development, metabolism, and response to stresses (Raffaele et al., 2008; Katiyar et al., 2012b; Ambawat et al., 2013). Zhang et al. (2019) stated that the MYB30 TF is involved in cuticular wax biosynthesis and resistance against pathogens. Wen et al. (2016) found that *CsDof* TF might play a role against biotic stresses in cucumber (*Cucumis sativus*). Rojas-Gracia et al. (2019) stated the SIDOF10 TF involved regulation of vascular tissue in tomatoes (*Solanum lycopersicum*). C2H2 TF regulates abiotic stress responses in plants (Han et al., 2020). TCP TFs regulate plant growth, development, and stress response (Li, 2015; Danisman, 2016). LBD TFs play role in anthocyanin and nitrogen metabolisms, pollen development, and pathogen response (Grimplet et al., 2017). NAC TF promoted cuticle wax biosynthesis in citrus (Yang H. et al., 2022). bHLH TF is involved in plant growth, metabolism, light signal transduction, cuticle development (Li et al., 2016; Sun et al., 2018). the qRT-PCR expression analysis was performed and found that the expression levels of *PeKCS* genes were positively or negatively regulated under stress conditions containing numerous TFs (Figures 12, 13). Our results are consistent with previous reports suggesting a role for *PeKCS* TFs in regulating of VLCFAs, wax biosynthesis, and plant stress responses, while wax regulation analysis requires further investigation.

Gene ontology and KEGG annotation results of *PeKCS* genes revealed that highly enriched GO terms were associated with fatty acid synthase and elongase activity, acyltransferase activity, endoplasmic reticulum, and response to stresses, while KEGG pathways included fatty acid elongation, lipid metabolism, and plant-pathogen interaction (Figure 9). Our GO results were consistent with qRT-PCR expression analysis, for example, *PeKCS2*, *PeKCS9*, and *PeKCS13* (Figure 12) were highly upregulated under drought stress conditions compared to controls, and these genes contained stress-responsive GO-BP terms (Supplementary Tables 13a,b). It has been reported that *KCS* genes take part in different processes including fatty acid elongase Todd et al. (1999), pathogen interaction (Xue et al., 2020), and lipid metabolism in the endoplasmic reticulum (De Bigault Du Granrut and Cacas, 2016). *KCS* genes have been reported to play important roles in VLCFA and wax biosynthesis as well as biotic and abiotic stresses (Kim et al., 2013; Lee and Suh, 2013; Lokesh et al., 2019; Yang Z. et al., 2020; Lian et al., 2021). The expression profiles of *KCS* genes in different tissues have been described earlier in numerous plant species and exhibited a diverse expression pattern among them. For example, in *C. sinensis*, Cs8g17800 and Cs4g04880 exhibited higher expression levels in flowers. Cs7g13310 and Cs7g28170 showed higher expression levels in the leaf. Cs7g04850 was expressed in flower and embryo. Cs4g17260 and Cs6g02360 were mainly expressed in flavedo. Cs2g16470 was universally expressed in the fruit. The expression levels of Cs2g16470, Cs8g17800, Cs4g17260, Cs6g02360, and orange1.1t00556 genes steadily increased along with fruit ripening (Yang H. et al., 2021).

In apple, *MdKCS*6 showed higher expression in leaves whereas, *MdKCS*1 was highly expressed in the peel. Overall, the highest expression levels were found in most of the *MdKCS* genes in apple peels (Lian et al., 2020). In *Arabidopsis KCS*6 and *KSC10* were highly expressed compared in roots, stems, flowers, and siliques. *KCS19* was not expressed in leaves and stems (Joubès et al., 2008). In the current study, the following genes exhibited higher expression patterns throughout the fruit developmental stages including *PeKCS*1 (FPKM = 17–55.62), *PeKCS*10 (FPKM = 13.73–324.52), and *PeKCS*30 (FPKM = 96.12–356.7) (Figure 11A). *PeKCS*20 showed the highest expression (FPKM = 213.63) in L conditioned root. *PeKCS* 29, showed the highest expression (FPKM = 1692.94) in the peel. *PeKCS*5 showed the highest expression (FPKM = 359.34) under NT and CS leaves (FPKM = 306.76, *PeKCS5*) (Figure 11B). *PeKCS*31 and *PeKCS*32 were not expressed in all pulp tissues, whereas four genes (*PeKCS*16, *PeKCS*17, *PeKCS*18, and *PeKCS*19) were not expressed in leaves, peel, and root tissues, indicating that they might not involve in passion fruit growth and development. These diverse expression results indicate that *PeKCS* genes have specific expression patterns in all tested tissues and may play important roles in specific functions, but further research is required.

Consistent with the findings of other studies, *KCS* has been reported to be involved in regulating plant responses to drought and biotic stresses (Li et al., 2018; Lokesh et al., 2019; Guo et al., 2020; Wang Y. et al., 2020). The expression patterns of *PeKCS* genes under drought stress and *F. kyushuense* fungal biotic stress conditions (Figures 12, 13) were analyzed. Most of the genes were positively or negatively regulated under stress conditions. Among them, *PeKCS*20 was up-regulated (29-fold) in yellow roots, *PeKCS*27 and *PeKCS*13 were up-regulated (8 to10-fold) in yellow and purple leaves under drought conditions. *PeKCS*1 was down-regulated in all tissues (root, stem, and leaves), indicating that its expression was inhibited by drought conditions. Relative expressions of *PeKCS*4 and *PeKCS*8 were up-regulated (4 and 3-folds) in yellow leaves but down-regulated in roots and stems (Figure 12). The expression profiles of *PeKCS* genes under biotic stress conditions (*F. kyushuense* fungal stress) showed that all *PeKCS* genes were expressed at different expression levels and most of the *PeKCS* genes were upregulated under biotic stress in purple 9th dpi except *PeKCS*1 compared to yellow 9th dpi (Figure 13). *PeKCS*20 was upregulated up to 9 folds, *PeKCS*2 > 6 folds, *PeKCS*9, *PeKCS*27, and *PeKCS*29 > 4 folds, *PeKCS*8 > 2.5-fold, *PeKCS*4 and *PeKCS*13 > 1-fold at 9th.dpi in purple cultivar. Whereas in yellow cultivar, *PeKCS*8, *PeKCS*2 and *PeKCS*20 were upregulated > 1- 3folds at 9th dpi. Only two gens *PeKCS*1 and *PeKCS*28 were upregulated > 1–2 folds at 12th dpi in the yellow cultivar.

Similar results were found by Lian et al. (2020) in apple *MdKCS* genes, eight *MdKCS* genes showed fluctuated (down-up-down-regulation) trend under drought conditions. *MdKCS*12 and *MdKCS*24 showed upregulated expressions (5 to 10-folds) under drought conditions. *MdKCS*6 expression was downregulated most of the time under drought conditions. The expressions of *KCS20* and *KCS2*/*DAISY* genes were upregulated in *Arabidopsis* stem under drought stress (Lee et al., 2009). *HvKCS*1 improved the barley leaf waxes and resistance to barley powdery mildew fungus (Li et al., 2018). Previous reports indicate that the accumulation of cuticular wax and VLCFAs increases under stress conditions (Seo et al., 2011; Budke and Goffinet, 2016; Wang Y. et al., 2020). Our expression results suggest that positive or negative regulations of *PeKCS* genes may be due to the increase or decrease of cuticle wax and VLCFAs under drought and fungal stress conditions, but further studies are required.

## Conclusion

In this study, comprehensive analyses were performed and identified 32 *PeKCS* genes in the passion fruit genome. All identified *PeKCS* genes were subjected to physio-chemical features, evolutionary relationships, motifs, gene structures, *cis*-regulatory elements, protein–protein interaction, syntenic analysis, TFs regulatory network analysis, GO, KEGG annotation, and putative micro-RNAs prediction analysis. The *KCS* gene family was expanded and subjected to purification screening. The hypothetically predicted subcellular localization of *PeKCS* genes was validated by a transient expression assay of *the PeKCS2* gene in the onion epidermis cell. Plant TFs families including ERF, MYB, and Dof were identified, and a regulatory network associated with the *PeKCS* genes was performed. FPKM-based gene expression profiles of *PeKCS* exhibited a diverse expression in the root, peel, leaves, and pulp tissues. The qRT-PCR based expressions suggested that most of the *PeKCS* genes were highly upregulated in leaves under drought and at 9th dpi under fungal (*F. kyushuense*) stresses conditions, specifically for genotype compared to controls. These findings provide a basis for future studies on the functions of *KCS* genes in passion fruit. Attempting to breed passion fruit cultivars susceptible to biotic or abiotic stress by overexpressing or inhibiting *KCS* gene expression in passion fruit tissues may provide new clues for the selection of passion fruit resistant cultivars. In future studies, we will further explore the role of *PeKCS* genes in VLCFA and cuticle wax biosynthesis on transcriptomics and metabolomic levels of passion fruit.

## Data Availability Statement

The original contributions presented in the study are included in the article/Supplementary Material, further inquiries can be directed to the corresponding author/s.

## Author Contributions

FC and HMR convinced the idea. HMR, FS, XL, AFY, MS, QY, YC, and KS performed the formal analysis and qRT-PCR. MB, HMR, and S-YH performed the bioinformatics analysis and wrote the manuscript. FC, RO, MYMJ, MA, MAAA, ZM, and LZ revised and edited the manuscript. All authors have read and agreed to the published version of the manuscript.

## Conflict of Interest

The authors declare that the research was conducted in the absence of any commercial or financial relationships that could be construed as a potential conflict of interest.

## Publisher’s Note

All claims expressed in this article are solely those of the authors and do not necessarily represent those of their affiliated organizations, or those of the publisher, the editors and the reviewers. Any product that may be evaluated in this article, or claim that may be made by its manufacturer, is not guaranteed or endorsed by the publisher.

## Footnotes

1
https://ftp.cngb.org/pub/CNSA/data3/CNP0001287/CNS0275691/CNA0017758/

2
http://www.Arabidopsis.org

3
https://iris.angers.inra.fr/gddh13/the-apple-genome-downloads.html

4
https://www.peanutbase.org/download

5
http://pfam.xfam.org/

6
https://www.ncbi.nlm.nih.gov/Structure/bwrpsb/bwrpsb.cgi

7
http://smart.embl-heidelberg.de/

8
https://web.expasy.org/protparam/

9
http://cello.life.nctu.edu.tw

10
https://www.megasoftware.net/

11
https://itol.embl.de/login.cgi

12
http://imed.med.ucm.es/Tools/sias.html

13
http://meme-suite.org/index.html

14
http://bioinformatics.psb.ugent.be/webtools/plantcare/html/

15
https://string-db.org/cgi/input.pl

16
https://npsa-prabi.ibcp.fr/cgi-bin/npsa_automat.pl?page=npsa_sopma.html

17
http://www.sbg.bio.ic.ac.uk/phyre2/html/

18
https://www.zhaolab.org/psRNATarget/

19
https://cytoscape.org/download.html

20
http://eggnog-mapper.embl.de/

21
http://plantregmap.gao-lab.org/binding_site_prediction.php

22
http://www.ncbi.nlm.nih.gov/sra

23
https://ubuntu.com/wsl

24
https://www.graphpad.com/scientific-software/prism
