# Supplementary material for: Genome-Wide Identification and Expression Profiling of KCS Gene Family in Passion Fruit (Passiflora edulis) Under Fusarium kyushuense and Drought Stress Conditions
Source: Front Plant Sci. 2022 Apr 25;13:872263. doi: 10.3389/fpls.2022.872263 (PMC9081883; doi:10.3389/fpls.2022.872263)
Supplement: Supplementary file 1 [file Data_Sheet_1.ZIP › Supplementary Table S4.docx]

**Supplementary Table S4.** Putative motifs identified from PeKCS proteins using MEME. The sequence logos were generated using WebLogo.

| **Motif name** | **Sequence logo** | **E-value** |
| --- | --- | --- |
| **Motif 1** | 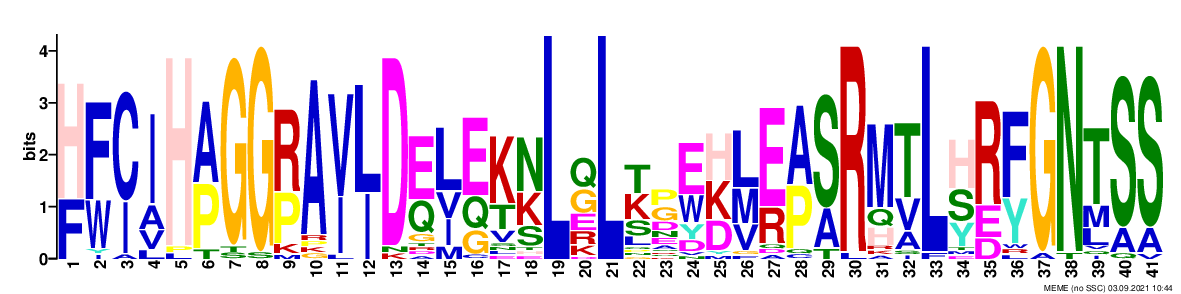  HFCIHAGGRAVJDELEKNLQLTPEHLEASRMTLHRFGNTSS | 6.5e-673 |
| **Motif 2** | 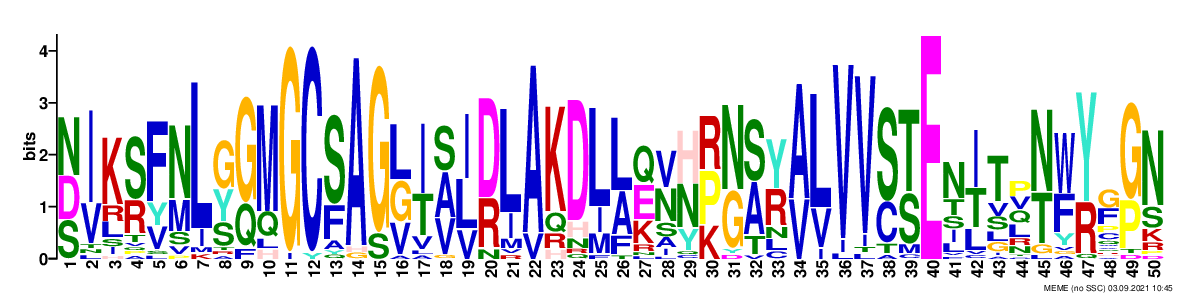  NIKSFNLGGMGCSAGLIAJDLAKDLLQVHRNSYALVVSTENITPNWYGGN | 4.3e-711 |
| **Motif 3** | 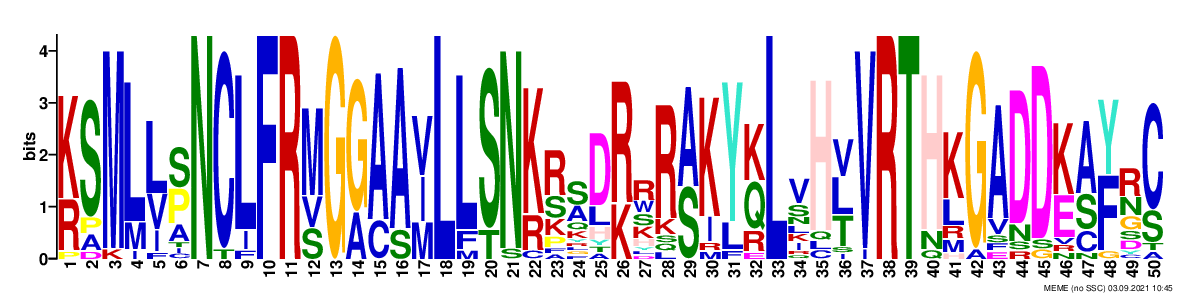  KSMLLSNCLFRMGGAAVLLSNKRSDRRRAKYKLVHVVRTHKGADDKAYRC | 5.6e-526 |
| **Motif 4** | 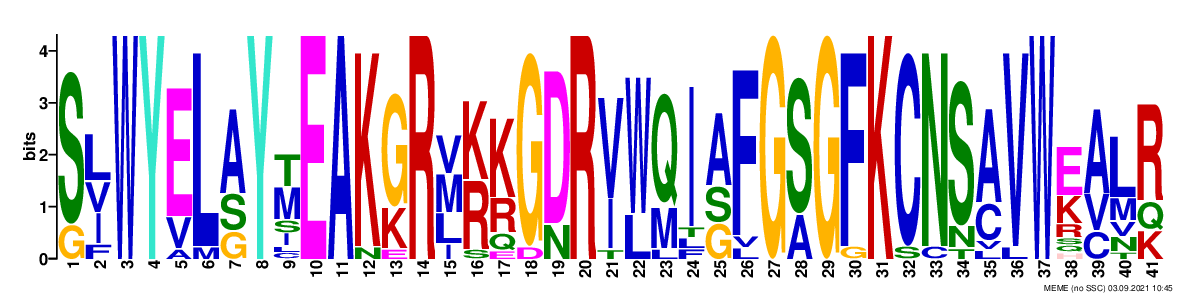  SLWYELAYTEAKGRVKKGDRVWQIAFGSGFKCNSAVWEALR | 1.3e-419 |
| **Motif 5** | 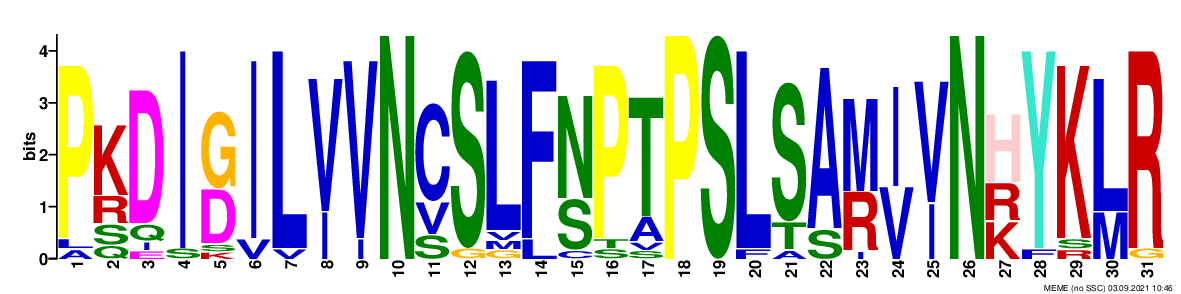  PKDIGILVVNCSLFNPTPSLSAMIVNHYKLR | 8.3e-369 |
| **Motif 6** | 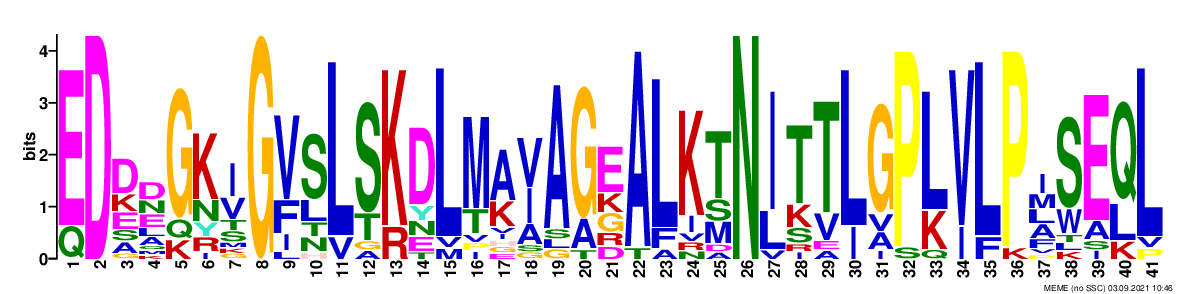  EDDDGKIGVSLSKDLMAVAGEALKTNITTLGPLVLPISEQL | 5.3e-274 |
| **Motif 7** | 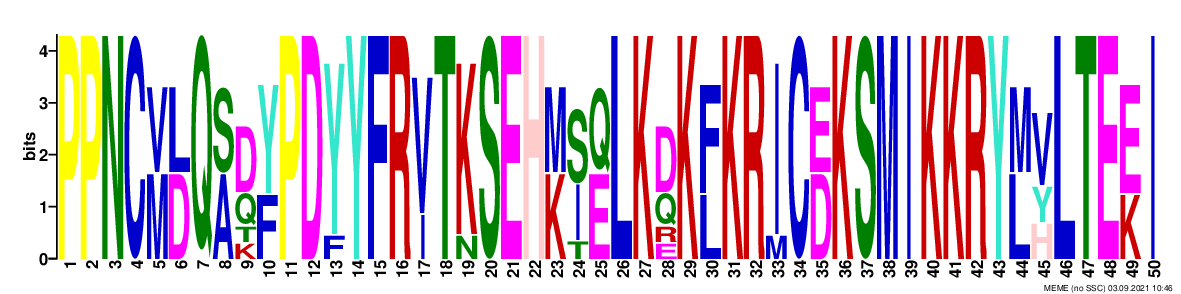  PPNCVLQADYPDYYFRVTKSEHKSZLKDKFKRICEKSMIKKRYLVLTEEI | 4.0e-261 |
| **Motif 8** | 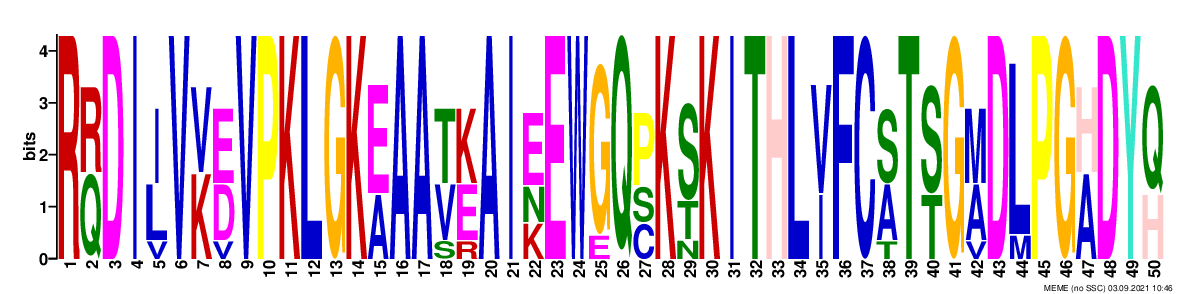  RRDIIVKEVPKLGKEAATKAIEEWGQPKSKITHLVFCSTSGMDLPGADYQ | 4.0e-237 |
| **Motif 9** | 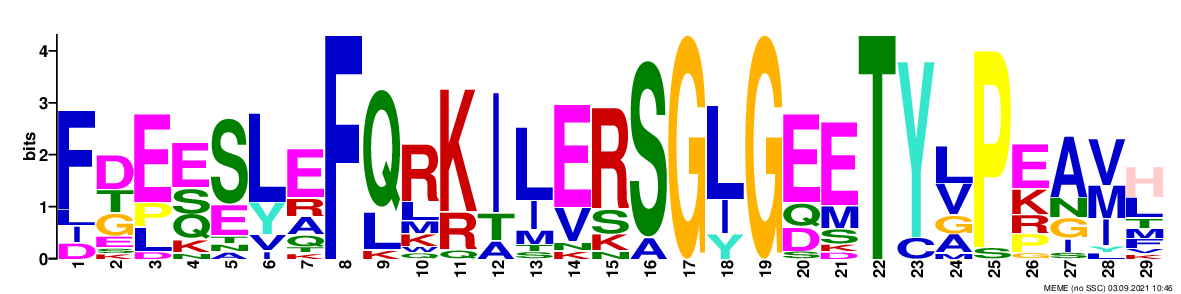  FDEESLEFQRKILERSGLGEETYLPEAVH | 3.3e-206 |
| **Motif 10** | 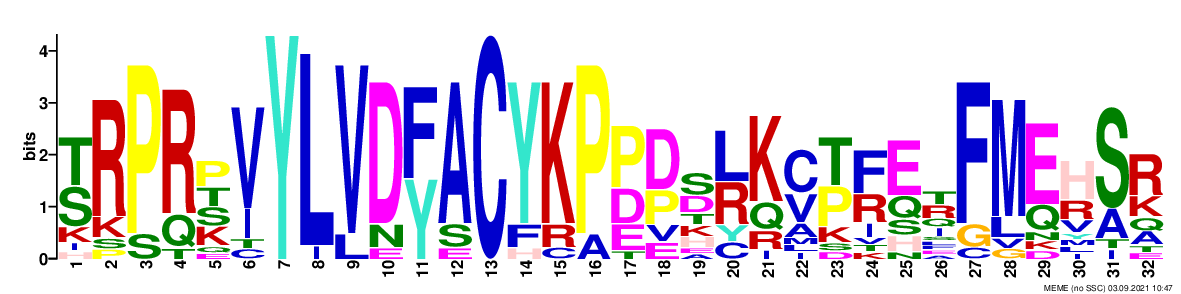  TRPRPVYLVDFACYKPPDSLKCTFETFMEHSR | 2.1e-189 |
