# Supplementary material for: Genome-Wide Identification and Expression Profiling of KCS Gene Family in Passion Fruit (Passiflora edulis) Under Fusarium kyushuense and Drought Stress Conditions
Source: Front Plant Sci. 2022 Apr 25;13:872263. doi: 10.3389/fpls.2022.872263 (PMC9081883; doi:10.3389/fpls.2022.872263)
Supplement: Supplementary file 1 [file Data_Sheet_1.ZIP › Supplementary Figure S1.docx]

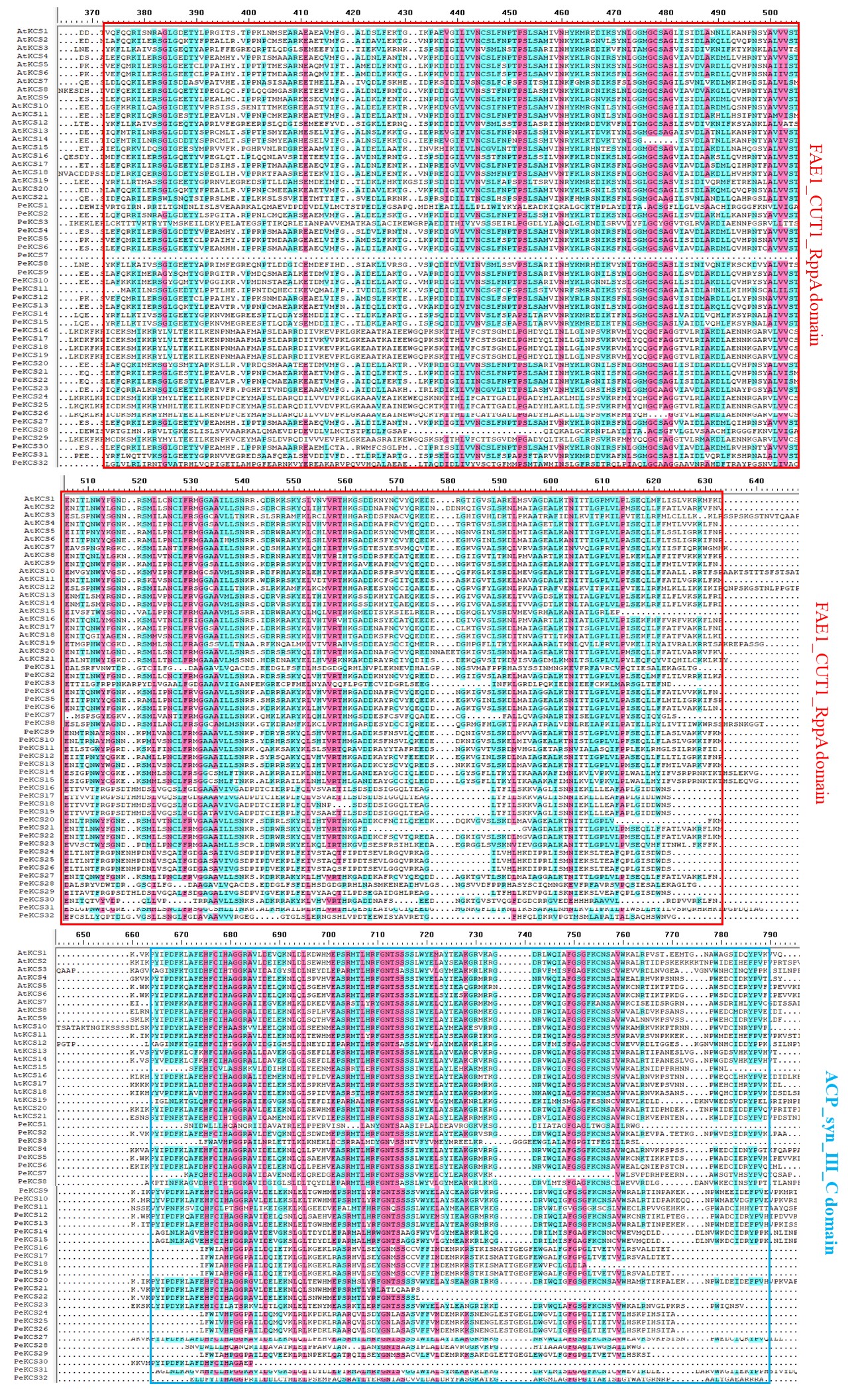


**Supplementary Figure S1.** Multi-sequence alignment and domain analysis of *AtKCS* and *PeKCS* proteins. Red box represents the FAE1_CUT1-RppA domain and blue box represents the ACP_syn_III_C domain. (Readers are referred to the Web version of this article for better understanding of the references to color in this figure legend)
