# Supplementary material for: Genome-Wide Identification and Expression Profiling of KCS Gene Family in Passion Fruit (Passiflora edulis) Under Fusarium kyushuense and Drought Stress Conditions
Source: Front Plant Sci. 2022 Apr 25;13:872263. doi: 10.3389/fpls.2022.872263 (PMC9081883; doi:10.3389/fpls.2022.872263)
Supplement: Supplementary file 1 [file Data_Sheet_1.ZIP › Supplementary Table S1.docx]

| **Supplementary Table1 S1. Primer sequences of *PeKCS* genes used in qRT-PCR.** | | |
| --- | --- | --- |
| **Gene** | **Forward primer (5' -3')** | **Reverse primer (5' -3')** |
| ***PeKCS*1** | AGATCATGCATTGGGTCCCA | CGCATCGAACAGCAAAACGA |
| ***PeKCS2*** | TTTCAGGATGGGCTGCTCAG | TGAAACTGCGATCATCGGCT |
| ***PeKCS4*** | GCAGCTGTTGTTCTTTGCGA | ACCGGCGTGTATGCAGAAAT |
| ***PeKCS8*** | AGAAGATGAACAAGGGCGCA | AGCTCTGTTGCTGGCAAGAT |
| ***PeKCS9*** | ACCAAATCACCCCAACCCTT | AGCGCATATCAATGGCAGGA |
| ***PeKCS13*** | ATCGACCTTGCCAAGCAACT | AGGCAGTTCGAGACAAGCAT |
| ***PeKCS20*** | TCGTCCCAGGCAAGTTTACT | AAGCTTTCCTCCGTGAAGCA |
| ***PeKCS27*** | AGGCAGAGCAGGTGATGTTT | AGCGATGGTGTGGGATTGAA |
| ***PeKCS28*** | TTGCTGTTCGATCTGTGCCT | TGCAACCGCATCAATGATCC |
| ***PeKCS29*** | AAACAACAAAGGCGCTCGTG | CCGCACCATCACTGAACAAT |
| **Pe-60S** | AGGTGGGTAACAGGATTATC | TGGCTGTCTTTTGGTGCTG |
